# A novel protein encoded by porcine circANKRD17 activates the PPAR pathway to regulate intramuscular fat metabolism

**DOI:** 10.1186/s40104-025-01153-5

**Published:** 2025-02-05

**Authors:** Xiao He, Fang Xie, Ying Nie, Xuefeng Wang, Junyi Luo, Ting Chen, Qianyun Xi, Yongliang Zhang, Jiajie Sun

**Affiliations:** Guangdong Provincial Key Laboratory of Animal Nutrition Control, National Engineering Research Center for Breeding Swine Industry, State Key Laboratory of Swine and Poultry Breeding Industry, College of Animal Science, South China Agricultural University, Guangzhou, Guangdong 510642 China

**Keywords:** CircRNAs, Intramuscular fat, Meat quality, PPAR pathway

## Abstract

**Background:**

Intramuscular fat is an important factor in evaluating pork quality and varies widely among different pig breeds. However, the regulatory mechanism of circular RNAs (circRNAs) in lipid metabolism remains largely unexplored.

**Results:**

We combined circRNA-seq and Ribo-seq data to screen a total of 18 circRNA candidates with coding potential, and circANKRD17 was found to be significantly elevated in the longissimus dorsi muscle of Lantang piglets, with a length of 1,844 nucleotides. Using single-cell sequencing, we identified 477 differentially expressed genes in IMF cells between Lantang and Landrace piglets, with enrichment in the PPAR signaling pathway. These genes included *FABP4*, *FABP5*, *CPT1A*, and *UBC*, consistent with the high levels of acylcarnitines observed in the longissimus dorsi muscles of the Lantang breed, as determined by lipidomic analysis. Further in vitro and in vivo experiments indicated that circANKRD17 can regulate lipid metabolism through various mechanisms involving the PPAR pathway, including promoting adipocyte differentiation, fatty acid transport and metabolism, triglyceride synthesis, and lipid droplet formation and maturation. In addition, we discovered that circANKRD17 has an open reading frame and can be translated into a novel 571-amino-acid protein that promotes lipid metabolism.

**Conclusions:**

Our research provides new insights into the role of protein-coding circANKRD17, especially concerning the metabolic characteristics of pig breeds with higher intramuscular fat content.

**Supplementary Information:**

The online version contains supplementary material available at 10.1186/s40104-025-01153-5.

## Introduction

Intramuscular fat (IMF) is a key indicator for evaluating pork quality [1] as it is closely linked to sensory qualities, such as color, flavor, tenderness [2], and marbling [3]. In general, the IMF content varies among different pig breeds [4]. Compared to western commercial pigs, Chinese local pigs have higher IMF content and better meat quality [5]. Specifically, Lantang (LT, a typical fatty pig breed) has fine marbling and high IMF, resulting in significantly superior meat quality compared to the Landrace (LW, a typical lean meat breed) [6]. In addition, the physiology and structure of pigs are similar to those of humans, making them excellent models for studying fat metabolism [7], thereby enabling more in-depth research into related diseases such as human obesity and insulin resistance [8].

In recent, several studies indicate that non-coding RNAs (ncRNAs), including microRNAs (miRNAs), long non-coding RNAs (lncRNAs), and circRNAs, are potential regulators of IMF, participating in the regulation of animal fat deposition processes [9]. In general, circRNAs are covalently closed RNA molecules generated through a process known as back-splicing. They are widely present in eukaryotic cells without a 5′ cap structure or 3′ polyA tail [10] and are composed of one or multiple exons [11]; circRNAs have long been considered functional RNAs that directly participate in various biological processes [12]. Previously, circRNAs were primarily studied for their non-coding functions, such as acting as miRNA sponges [13], participating in parental gene transcription [14], and interacting with proteins [15]. However, recent studies have found that circRNAs can directly code for proteins, as many of them contain translatable open reading frames (ORFs) [16]. Most endogenous circRNAs rely on Internal Ribosome Entry Sites (IRES) and N^6^-methyladenosine (m^6^A) modifications to initiate cap-independent translation mechanisms [17]. Despite this, the role of circRNA-encoded proteins in lipid metabolism remains largely unexplored.

In our study, we combined our previous circRNA-seq data [18] with Ribo-seq data [19] to evaluate the coding potential of circRNAs. We also investigated the functions and mechanisms of the circANKRD17 candidate in fat metabolism. These findings could lead to advancements in animal breeding strategies to improve meat quality and may also have implications in studying obesity-related conditions in humans.

## Materials and methods

### Ethics statement and animal tissue samples

A total of 20 healthy, purebred LT and LW pigs, 10 of each breed, were obtained post-birth from Banling Breeding Farm (Xinfeng County, Shaoguan City, Guangdong Province, China). The longissimus dorsi muscle tissues were collected and immediately snap-frozen in liquid nitrogen for lipid metabolomics and RT-qPCR analysis.

### Prediction of potential coding circRNA candidates with ribosome profiling

We utilized circRNA candidates obtained from our previous rRNA-depleted and RNase R-digested libraries [18] and predicted using CIRCexplorer2 [20]. For ribosome profiling data [19], the sequencing reads were filtered to remove mitochondrial, ribosomal RNA, and tRNA sequences and were subsequently aligned to the porcine reference genome. The unmapped reads, potentially containing junction reads of circRNAs, were collected to identify ribosome-associated circRNA candidates. We first extracted the exonic sequences surrounding the backsplice junction (50 bp on either side) and aligned all unmapped ribosome profiling reads (Ribo-seq reads that could not be aligned to the linear transcriptome or genome) to the circRNA backsplice junctions (100 bp sequences) using the BLASTn algorithm (https://blast.ncbi.nlm.nih.gov/Blast.cgi). We did not allow any mismatches and required a minimum read-junction overlap of 9 bp on either side of the junction. Furthermore, we developed a set of specific primers targeting the cyclization junction of these circRNA candidates. After reverse-transcribing total RNA into cDNA, this cDNA served as the template for subsequent PCR amplification. The head-to-tail splicing of circRNA was initially confirmed through 1% agarose gel electrophoresis and Sanger sequencing of the PCR products. The details of the primers used can be found in Table S1A.

### Characterization of IMF cells between LT and LW breeds

Based on our previous single-cell sequencing data, a total of 242 and 115 cells exhibited gene expression patterns that could be attributed to IMF cells in the LT and LW groups, respectively [21]. Using Seurat v4.0.3 [22], the “Normalization” function was employed to calculate gene expression levels, while the “FindAllMarkers” function was utilized to identify DEGs between LT and LW pigs with |log(Fold Change)| > 0.25 and a *P*-value < 0.05. The KEGG annotation of DEGs was performed using the “enrichKEGG” function in the clusterProfiler package v4.0.5 [23].

### Lipid metabolome determination and analysis in muscle tissue

The frozen longissimus dorsi muscle tissues were ground into a fine powder under liquid nitrogen using a pre-chilled mortar and pestle. For lipid extraction, ice-cold methanol and chloroform were added to the tissue samples at a ratio of 2:1 (v/v). The mixture was thoroughly vortexed and incubated on ice for 30 min to ensure efficient lipid solubilization. Following the incubation, chloroform and water were added to the samples to achieve a final solvent ratio of 1:1:0.9 (chloroform/methanol/water, v/v/v). The samples were then centrifuged at 4 °C for 10 min at 12,000 × *g* to facilitate phase separation. The lower organic phase, containing the lipids, was carefully collected using a glass pipette and transferred to a new tube for UHPLC-MS/MS analysis (LC-Bio Technology, Hangzhou, China). The raw mass spectrometry data were processed using proprietary software for peak detection, alignment, and integration. The peak intensities were then normalized to the total spectral intensity, and the normalized data were further used to predict the molecular formula based on additive ions, molecular ion peaks, and fragment ions. Next, the peaks were matched with the LipidMaps and LipidBlast databases to obtain accurate qualitative and relative quantitative results. Finally, the univariate analysis (*t*-test) was applied to calculate statistical significance (*P*-value). Metabolites with a VIP > 1, *P*-value < 0.05, and a fold change ≥ 2 or ≤ 0.5 were considered differential metabolites between LT and LW.

### Actinomycin D and RNase R treatment

The 3T3-L1 cells were seeded into 6-well plates. After reaching approximately 60% confluency at 24 h, the cells were treated with either 5 μg/mL Actinomycin D or DMSO (Sigma, St. Louis, MO, USA) and harvested at 6, 12, 18, and 24 h for further analysis. For RNase R treatment, around 2 μg of total RNA extracted from porcine muscle tissues was incubated with 3 U/μg of RNase R (Epicentre Technologies, Madison, WI, USA) for 15 min at 37 °C. Following RNase R treatment, the RNA was subjected to RT-qPCR analysis, including reverse transcription to cDNA, followed by the measurement of circRNAs and their host mRNAs expression levels using the RT-qPCR protocol.

### Nuclear and cytoplasmic extraction

The nuclear and cytoplasmic fractions of porcine muscle were separated using the PARIS™ Kit (Thermo Fisher Scientific, Waltham, USA). Samples were lysed on ice for 10 min, followed by centrifugation at 500 × *g* for 3 min at 4 °C. The supernatant was collected as the cytoplasmic fraction, while the pellet, containing nuclei, was washed and collected separately. Total RNA was then extracted from both fractions and analyzed by RT-qPCR to measure the levels of nuclear marker U6, cytoplasmic marker ACTB (encoding β-actin), and circANKRD17.

### Fluorescence in situ hybridization (FISH)

A 5′-Cy3-labeled probe specific to circANKRD17 was synthesized (Sangon Biotech, Shanghai, China). The experiment was carried out using a Fluorescence In Situ Hybridization Kit (RiboBio, Guangzhou, China). Probes targeting the reverse splice site of circANKRD17 were used to determine its subcellular localization. Images were captured with a Zeiss LSM 710 confocal microscope (Zeiss, Oberkochen, Germany).

### Vector construction and RNA oligonucleotides

The linear sequence of circANKRD17 was cloned into the pCD2.1-ciR vector (Geneseed Biotech, Guangzhou, China) and designated as OE-circANKRD17, with the empty pCD2.1-ciR vector serving as the negative control. Two small interfering RNAs (siRNAs), si-circANKRD17-1 and si-circANKRD17-2, targeting the junction sites of circANKRD17, along with a negative control siRNA, were designed and synthesized (GenePharma, Shanghai, China). Additionally, the 1716-nt ORF sequence within murine circANKRD17 was cloned into the pcDNA3.1 vector (Invitrogen, Carlsbad, CA, USA) with a 3 × Flag tag sequence inserted before the stop codon, referred to as pCDNA3.1-circANKRD17-Flag; the empty pcDNA3.1 vector was used as the corresponding negative control.

### Cell culture and treatment

Mouse immortalized 3T3-L1 preadipocytes were obtained from the China National Collection of Authenticated Cell Cultures (Beijing, China) and cultured at 37 °C in 5% CO_2_. Differentiation was induced 2 d after the cells reached confluence by supplementing the growth media with 3 nmol/L insulin, 0.25 µmol/L dexamethasone, and 0.5 mmol/L 1-methyl-3-isobutyl-xanthine (Beyotime Biotechnology, Shanghai, China). Then, the cells were transfected with the overexpression vector, knockdown vector, and their respective negative controls using Lipofectamine 2000 (Invitrogen). The transfected cells were harvested for protein and RNA analysis 48 h later.

### Oil red O, Nile red staining and triglyceride content assay

The medium of 3T3-L1 cells was removed, and the cells were fixed with 4% paraformaldehyde (Solarbio, Beijing, China) for 30 min. Oil red O staining solution (Sigma) was added for 1 h, or Nile red working solution (Sigma) was applied for 10 min. After rinsing with PBS, the staining results were observed and photographed under a microscope (Olympus, Tokyo, Japan). The triglyceride content was determined using a triglyceride test kit (Jiancheng Bioengineering Institute, Nanjing, China). The liver tissues of the tested mice were fixed with paraformaldehyde, dehydrated in graded alcohol, washed in xylene, and then paraffin-embedded for sectioning. Sections of 5 μm thickness were cut longitudinally using a manual rotary slicer (Olympus, Tokyo, Japan). The sections were then stained with Oil red O and scanned.

### Protein isolation and western blot

Total proteins were extracted from 3T3-L1 cells or gastrocnemius muscle using RIPA lysis buffer (Solarbio, Beijing, China). A total of 15 μg of protein was subjected to SDS-PAGE and subsequently transferred onto polyvinylidene fluoride (PVDF) membranes (Millipore, Bedford, MA, USA). The membranes were then probed with specific primary antibodies, followed by incubation with goat anti-rabbit IgG-HRP or goat anti-mouse IgG-HRP secondary antibodies (Bioworld, Minneapolis, MN, USA). Immunoreactive protein bands were visualized using an enhanced chemiluminescence solution (Solarbio). The antibodies used targeted proteins including PPARγ, CEBPα, CD36, FABP4, FABP5, FASN, LPL, and CPT1A.

### Statistical analysis

Differences between the two groups were analyzed using ANOVA followed by a Student’s *t*-test. Statistical significance was set at *P* < 0.05, with significance levels indicated by one asterisk for *P* < 0.05 and two for *P* < 0.01. Each experiment included at least three biological replicates.

## Results

### Identification of coding-potential circRNAs in porcine muscle tissues

**Fig. 1:**
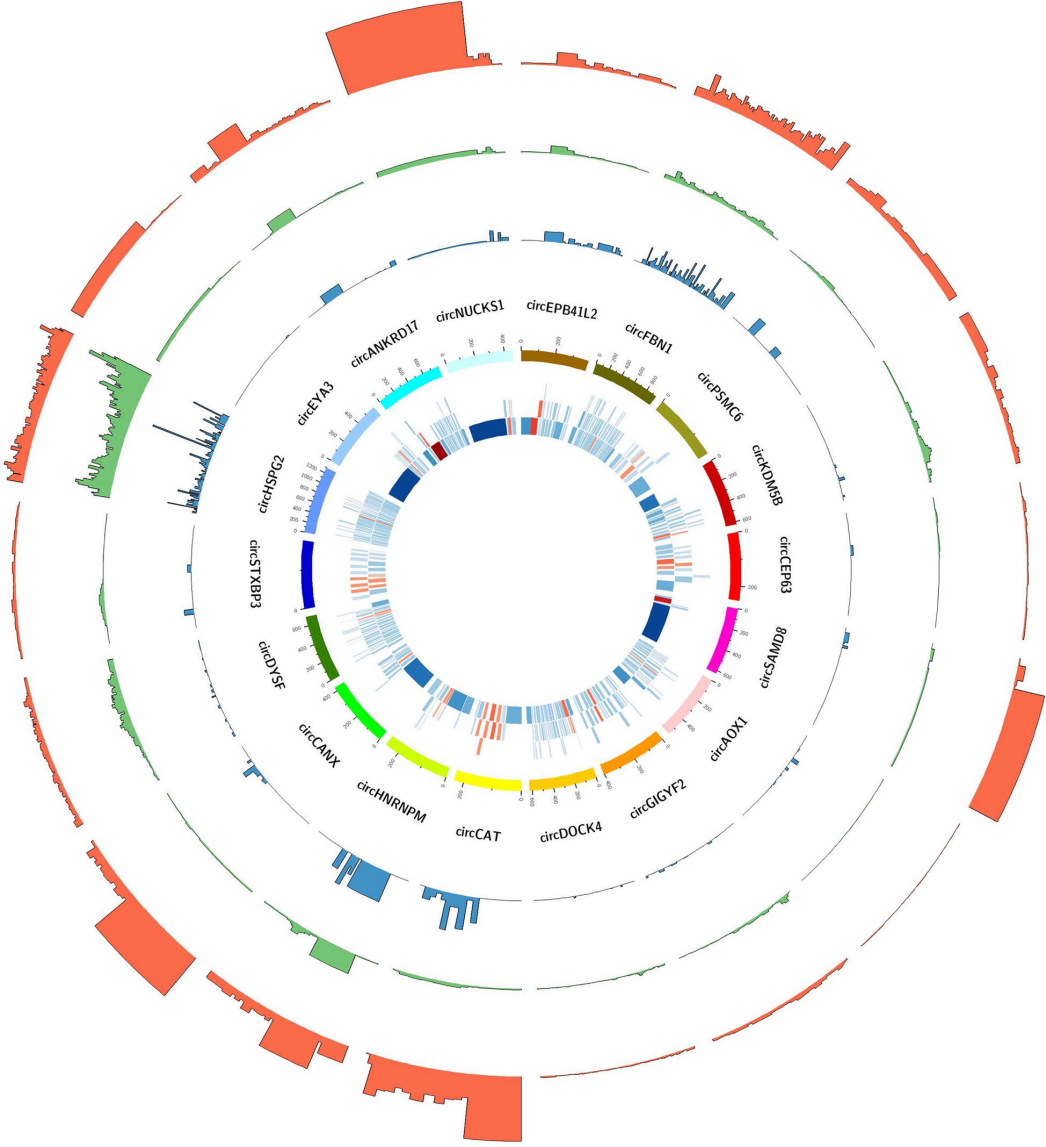
Identification of coding-Potential circRNAs in porcine muscle tissues. In the inner circle, the tiles represent the exons of host genes. Exons marked in red indicate circRNA exons, while those in blue represent non-circRNA exons. The darker the color, the larger the exon. In the second inner circle, the histogram displays the abundance of reads for each exon of the host genes in the Ribo-seq library. In the third inner circle, the histogram shows the abundance of reads for each exon of the host genes in the RNase R-digested libraries. In the outer circle, the histogram presents the abundance of reads for each exon of the host genes in the rRNA-depleted libraries

A total of 25,295 circRNA candidates were predicted by CIRCexplorer2 in the longissimus dorsi tissues of LT and LW piglets. Among these, 18 circRNAs with coding potential were identified by combining the ribosome profiling sequencing in porcine longissimus dorsi tissues (Table S1B). We then designed divergent primers based on the junction sequences of the circRNAs and successfully confirmed the circular characteristic of 8 circRNA candidates based on reverse transcription PCR (RT-PCR) and Sanger sequencing (Fig. S1A). Among them, circANKRD17, which consists of exon 28 and exon 29 of its parental gene, has a fragment size of 1,844-nt, while the sequencing reads was obviously concentrated in the exon 28–29 region in the rRNA removal, RNase R digestion, and Ribo-seq libraries (Fig. 1). In our study, only the expression level of circANKRD17 was significantly elevated in the longissimus dorsi muscle of LT pigs (Fig. S1B, *P* < 0.01), suggesting that circANKRD17 may be related to porcine intramuscular fat metabolism.

### Analysis of IMF cell characteristics and lipid metabolism differences between pig breeds

**Fig. 2:**
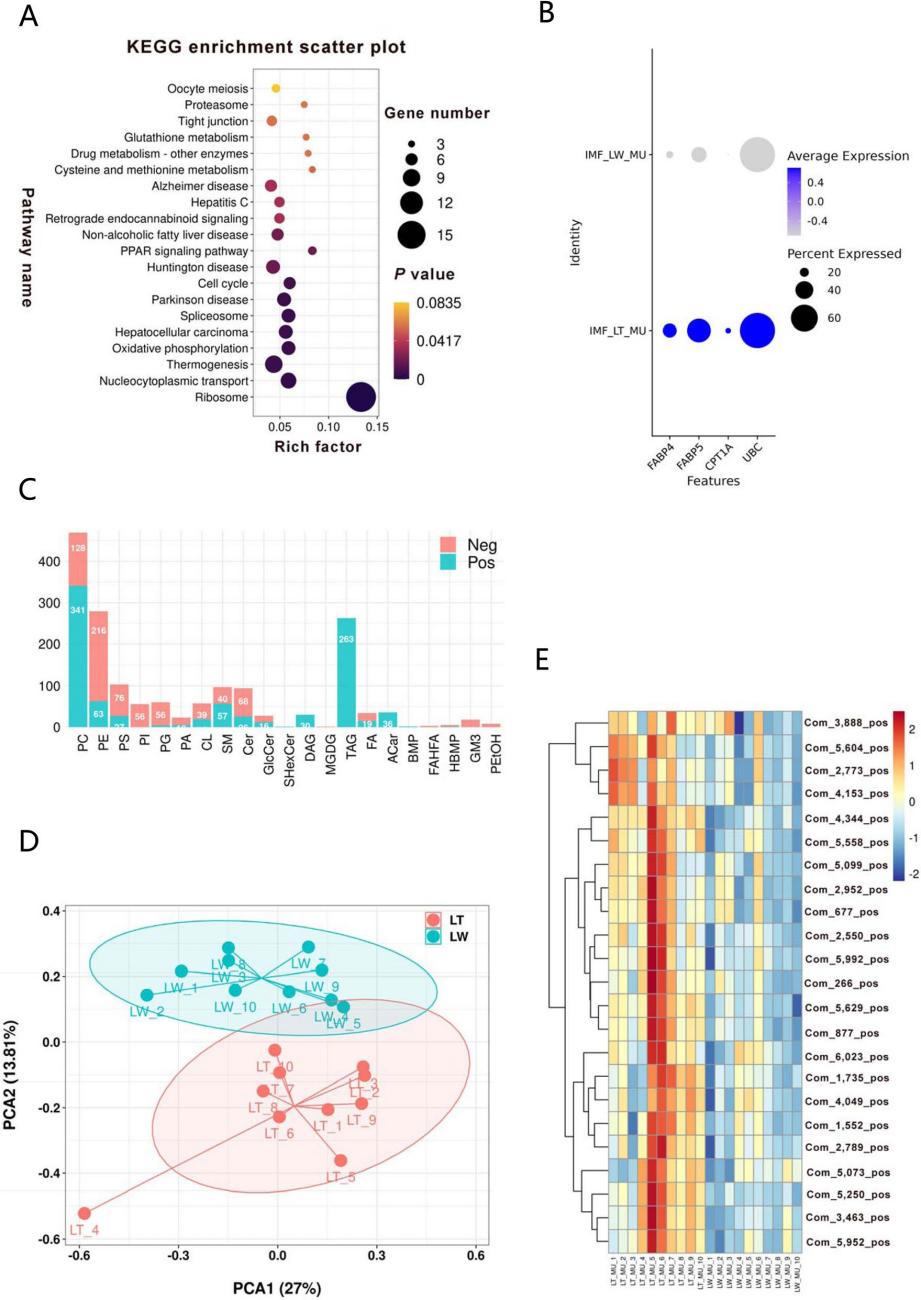
Characteristics of IMF cells and lipid metabolism differences between pig breeds. **A** KEGG pathway enrichment analysis of differentially expressed genes. **B** Expression levels of genes related to the PPAR pathway. **C** Fat metabolites identified in porcine serum. Abbreviations: PC, phosphatidylcholine; PE, phosphatidylethanolamine; PS, phosphatidylserine; PI, phosphatidylinositols; PG, phosphatidylglycerols; PA, phosphatidic acids; CL, cardiolipin; SM, sphingomyelins; Cer, ceramides; GlcCer, glucosyl ceramides; ShexCer, sulfated hexosyl ceramides; DAG, diacylglycerol; MGDG, monogalactosyldiacylglycerol; TAG, triacylglycerol; FA, free fatty acids; ACar, acylcarnitine; BMP, bis (monoacylglycerol) phosphate; FAHFAs, branched fatty acid esters of hydroxy fatty acids; GM3, monosialodihexosyl ganglioside; PetOH, phosphatidylethanol. **D** PCA scatter plot of lipid profiles for each test sample. Each point represents a sample, with differences between samples reflected in the separation and clustering trends in the plot. **E** Heatmap of differentially expressed acylcarnitines between LT and LW breeds

Based on our single-cell RNA data [21], a total of 477 differentially expressed genes were identified in IMF cells between LT and LW piglets. These candidates were enriched in the PPAR signaling pathway according to KEGG enrichment analysis (Fig. 2A). The pathway genes *FABP4*, *FABP5*, *CPT1A*, and *UBC* showed significantly higher expression levels in LT piglets (Fig. 2B). In general, FABPs are typically described as intracellular proteins that mediate lipid uptake and intracellular transport [24], while CPT1A, a key enzyme in mitochondrial fatty acid oxidation, influences the balance between lipolysis and liposynthesis [25]. Our findings may provide novel insights into the regulatory factor for higher intramuscular fat content observed in local pig breeds. We further utilized lipidomic technology and detected 944 negative and 1,113 positive lipid metabolites in porcine longissimus dorsi muscles (Table S1C), with significant enrichment in phosphatidylcholine (PC), phosphatidylethanolamine (PE), and triacylglycerol (TAG) (Fig. 2C). The PCA analysis demonstrated a clear distinction in metabolite composition between LT and LW breeds (Fig. 2D). A total of 229 metabolites were significantly regulated (*P* < 0.05), with 142 candidates increased and 87 decreased in the LT breed (Table S1D). In detail, a total of 23 acylcarnitines (ACar) were significantly upregulated in the LT breed (Fig. 2E), consistent with the high level of CPT1A expression, which catalyzes the conversion of fatty acids into acyl-carnitine in LT IMF cells.

### Characterization of circANKRD17 in adipocytes

**Fig. 3:**
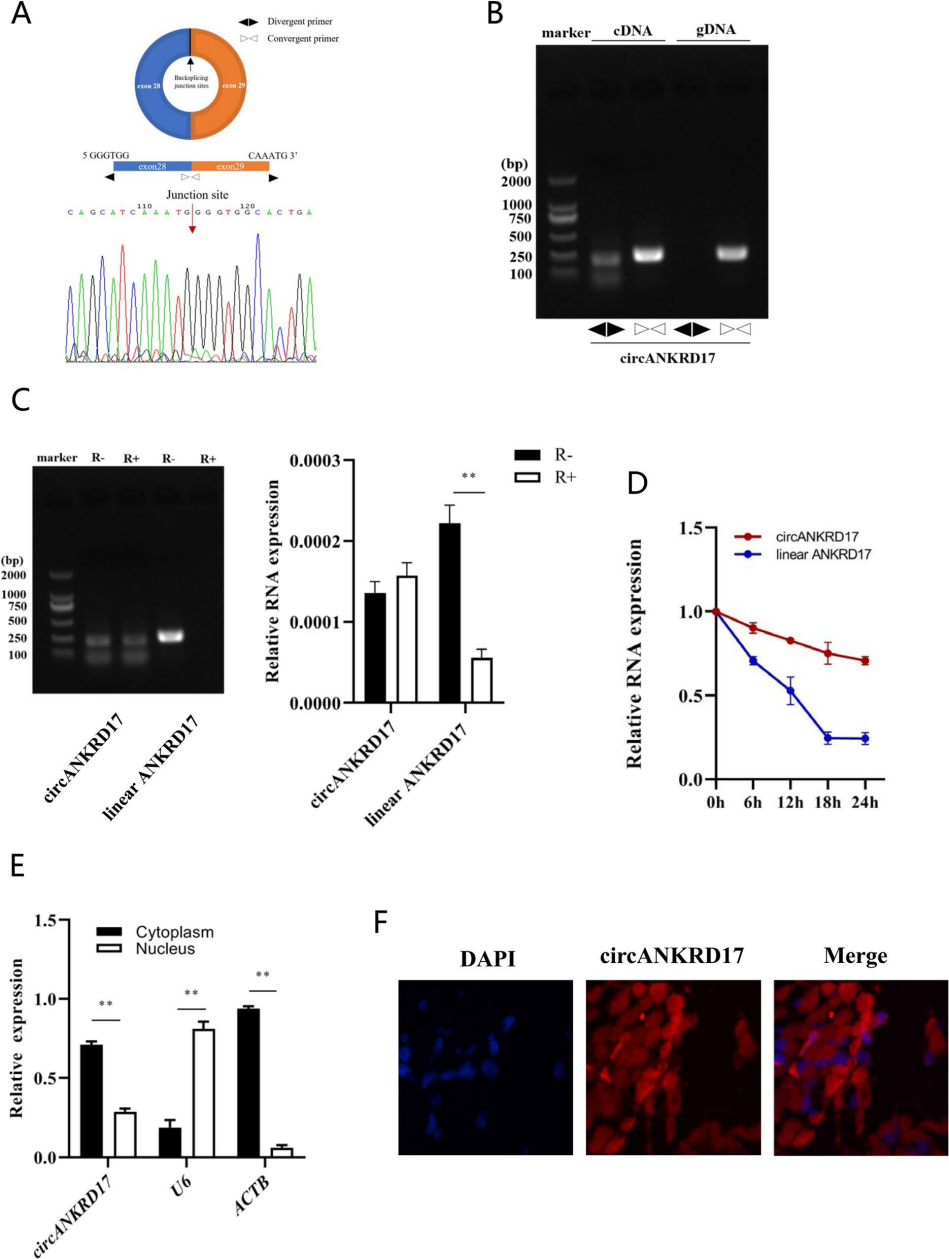
Characterization of circANKRD17 in adipocytes. **A** Circular structure diagram and Sanger sequencing results for circANKRD17. **B** Polymerase chain reaction (PCR) and agarose gel electrophoresis analysis of complementary DNA (cDNA) and genomic DNA (gDNA) using a double-primer method. **C** and **D** Analysis of circANKRD17 using RNase R digestion and actinomycin D treatment. circANKRD17 indicates a fragment with a junction sequence amplified by divergent primers, while linear ANKRD17 refers to a linear fragment amplified by convergent primers. **E** Distribution of circANKRD17 gene transcripts in 3T3-L1 cells, with U6 serving as a nuclear control gene and ACTB as a cytoplasmic control gene. **F** Localization of circANKRD17 observed via fluorescence in situ hybridization (FISH). Cy3-labeled circRNA appears red, while DAPI-stained nuclei appear blue. Scale bar = 10 μm

The head-to-tail splicing of circANKRD17 in mouse was successfully confirmed by RT-PCR and Sanger sequencing (Fig. 3A). The murine circANKRD17 contained a 1,653-nt open reading frame (Fig. S1C) and matched with the human hsa_circ_13463 from the circRNADb database (http://reprod.njmu.edu.cn/circrnadb) (Fig. S1D). This sequence spans from the putative ATG start codon to a STOP codon located 170-nt and 36-nt after the splice junction site, respectively. We used cDNA and genomic DNA (gDNA) isolated from 3T3-L1 cells as PCR templates, and divergent primers produced amplicons only from cDNA samples and not from the gDNA (Fig. 3B). Typically, linear RNA molecules are vulnerable to digestion by exonuclease R, whereas circRNA candidates exhibit resistance to this digestion. In our study, circANKRD17 was resistant to RNase R digestion compared with its linear parental gene based on agarose gel electrophoresis and RT-qPCR analysis (Fig. 3C). After treatment with Actinomycin D, a transcription inhibitor, RT-qPCR analysis revealed that the half-life of circANKRD17 exceeded 24 h, while the associated linear transcript had a half-life of approximately 4 h (Fig. 3D), indicating that circANKRD17 is more stable in 3T3-L1 cells. Further nuclear and cytoplasmic fractionation (Fig. 3E) and FISH (Fig. 3F) analyses revealed that circANKRD17 was primarily localized in the cytoplasm, as well as being present in the nucleus. These results collectively reveal that circANKRD17 is conserved across species and is expressed abundantly and stably in adipocytes.

### Overexpression and knockdown of circANKRD17 in lipid metabolism in vitro

**Fig. 4:**
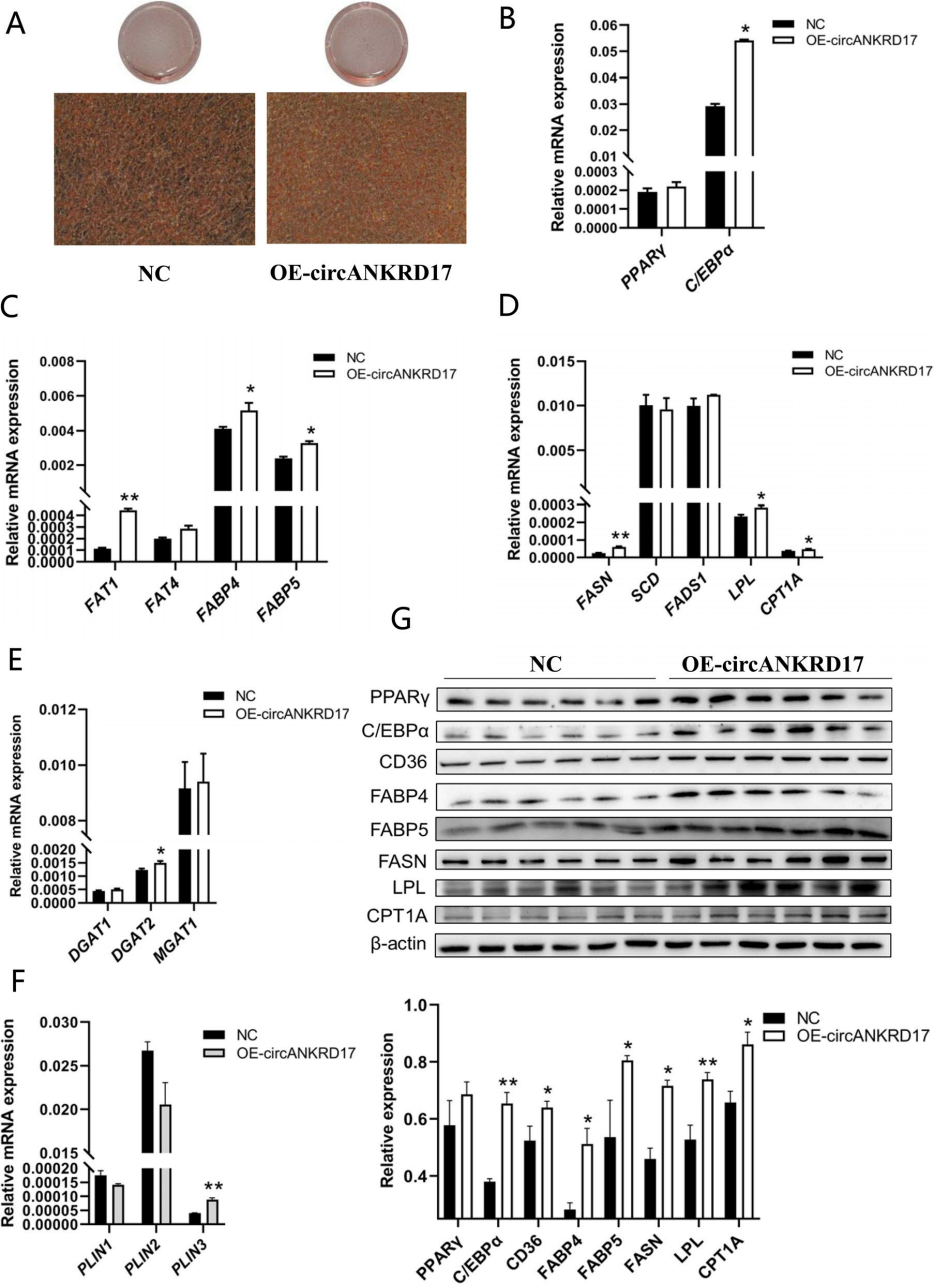
Overexpression of circANKRD17 promotes lipid metabolism in vitro. **A** 3T3-L1 cells stained with Oil red O after transfection with either NC (negative control) or OE-circANKRD17 (overexpression of circANKRD17). **B** The mRNA levels of adipocyte differentiation genes. **C** The mRNA levels of fatty acid transport genes. **D** The mRNA levels of fatty acid synthesis, hydrolysis, and oxidation genes. **E** The mRNA levels of triglyceride synthesis genes. **F** The mRNA levels of lipid droplet maturation genes. **G** Protein levels related to lipid metabolism after treatment with either the negative control or OE-circANKRD17

**Fig. 5:**
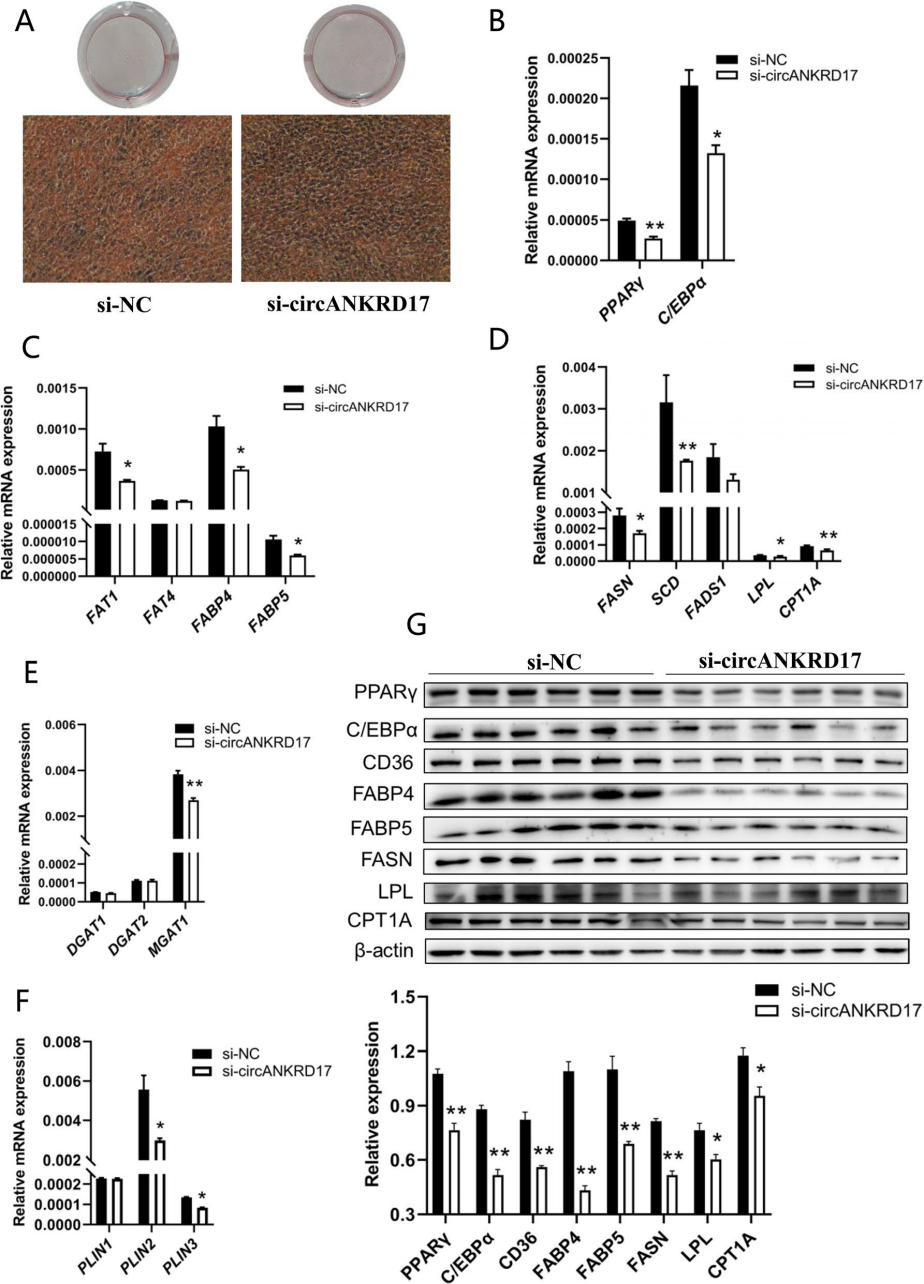
Knockdown of circANKRD17 inhibits lipid metabolism in vitro. **A** 3T3-L1 cells transfected with si-NC (negative control) or si-circANKRD17 (small interfering RNA targeting circANKRD17) and stained with Oil red O. **B** The mRNA levels of adipocyte differentiation genes. **C** The mRNA levels of fatty acid transport genes. **D** The mRNA levels of genes involved in fatty acid synthesis, hydrolysis, and oxidation. **E** The mRNA levels of triglyceride synthesis genes. **F** the mRNA levels of lipid droplet maturation genes. **G** Protein levels related to lipid metabolism after treatment with either si-NC or si-circANKRD17

We amplified the 1,844-nt full-length fragment of murine circANKRD17 (Fig. S1E) and constructed a recombinant plasmid (OE-circANKRD17) using the circRNA overexpression vector pCD2.1-ciR (Fig. S1F and Fig. S1G). In addition, we synthesized two siRNA fragments targeting the splice junction (si-circANKRD17-1 and si-circANKRD17-2). RT-qPCR showed that transfection of OE-circANKRD17 and si-circANKRD17-1 significantly increased and decreased the expression levels of circANKRD17 in 3T3-L1 cells (*P* < 0.01), respectively, with no significant effects on the host gene (Fig. S1H and Fig. S1I). After transfection with OE-circANKRD17, Oil red O staining revealed a obviously increase in the number of lipid droplets (Fig. 4A), while Nile red staining showed a substantial rise in triglyceride levels and content (Fig. S1J). The RT-qPCR results demonstrated that overexpression of OE-circANKRD17 significantly increased the mRNA expression levels of *CEBPα* involved in adipocyte differentiation (Fig. 4B, *P* < 0.05), *FAT1*, *FABP4*, and *FABP5* involved in fatty acid transport (Fig. 4C, *P* < 0.01 or *P* < 0.05), *FASN*, *LPL*, and *CPT1A* involved in fatty acid synthesis, hydrolysis, and oxidation (Fig. 4D, *P* < 0.01 or *P* < 0.05), *DGAT2* involved in intracellular triglyceride synthesis (Fig. 4E, *P* < 0.05), and *PLIN3* involved in lipid droplet formation and maturation (Fig. 4F, *P* < 0.01). In similar, OE-circANKRD17 significantly increased the protein expression levels of PPARγ, CEBPα, CD36 (FAT), FABP4, FABP5, FASN, LPL, and CPT1A (Fig. 4G, *P* < 0.01 or *P* < 0.05). We selected si-circANKRD17-1 for the follow-up knockdown experiments. In contrast, circANKRD17 knockdown effectively reduced lipid droplet production (Fig. 5A). Nile red staining and triglyceride content assays indicated a decrease in triglyceride levels (Fig. S1K). RT-qPCR analysis showed that si-circANKRD17-1 knockdown significantly suppressed the mRNA expression of *PPARγ* and *CEBPα* (Fig. 5B, *P* < 0.01 or *P* < 0.05), as well as *FAT1*, *FABP4*, *FABP5* (Fig. 5C, *P* < 0.05). The mRNA levels of *FASN*, *SCD*,* LPL*, and *CPT1A* were also significantly reduced (Fig. 5D, *P* < 0.01 or *P* < 0.05). In similar, *MGAT1* (Fig. 5E, *P* < 0.01) and *PLIN2*, *PLIN3* (Fig. 5F, *P* < 0.05) mRNA levels were significantly down-regulated. Western blot results confirmed that circANKRD17 knockdown significantly reduced the protein expression levels of PPARγ, CEBPα, CD36 (FAT), FABP4, FABP5, FASN, LPL, and CPT1A (Fig. 5G, *P* < 0.01 or *P* < 0.05). Our results suggest that circANKRD17 plays an important role in various aspects of lipid metabolism in vitro.

### Analysis of circANKRD17 in lipid metabolism in vivo

**Fig. 6:**
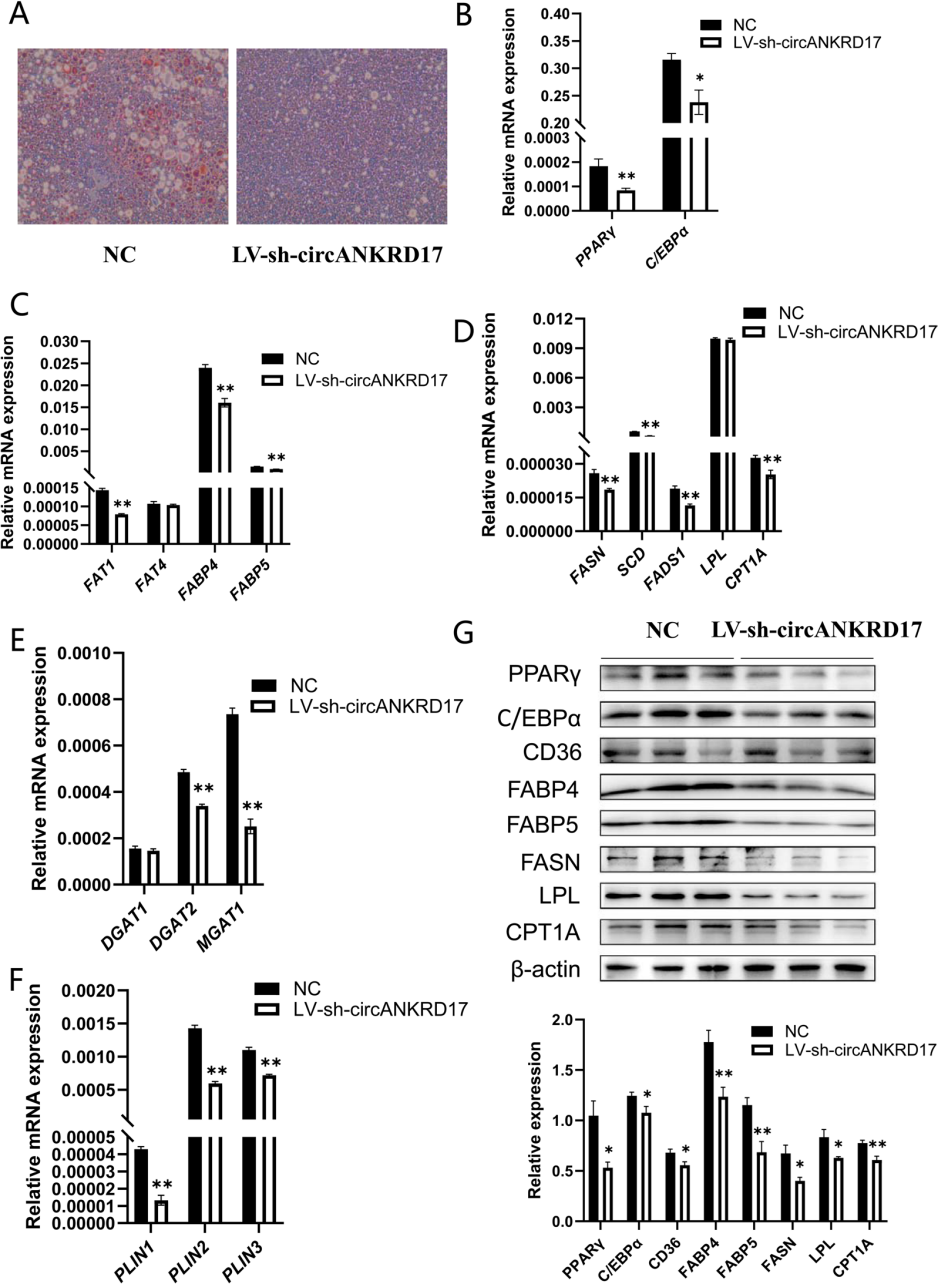
Knockdown of circANKRD17 inhibits lipid metabolism in vivo. **A** Oil red O staining of mouse livers infected with either LV-sh-circANKRD17 lentivirus or control, with high-fat diet treatments, scanned at 20 × magnification. **B** The mRNA levels of adipocyte differentiation genes. **C** The mRNA levels of fatty acid transport genes. **D** The mRNA levels of genes involved in fatty acid synthesis, hydrolysis, and oxidation. **E** The mRNA levels of triglyceride synthesis genes. **F** The mRNA levels of lipid droplet maturation genes. **G** Protein levels related to lipid metabolism after treatment with either the negative control (NC) or LV-sh-circANKRD17

We constructed a lentiviral vector encoding a short hairpin RNA (circANKRD17-si-1), referred to as LV-sh-circANKRD17 (Fig. S1L–M). Both LV-sh-circANKRD17 and an empty vector (NC) were injected into six 6-week-old C57BL/6 mice via tail vein injection. The first injection was administered on day 0, followed by weekly injections for a total of four doses. During the injection period, the mice were fed either a standard diet or a high-fat diet. Liver and gastrocnemius muscle samples were subsequently collected. In the standard diet groups, Oil red O staining demonstrated that no significant changes in lipid metabolism were observed in the livers between the LV-sh-circANKRD17 and NC groups (Fig. S1O). In the high-fat diet groups, RT-qPCR analysis revealed that LV-sh-circANKRD17 significantly reduced the expression of circANKRD17 in the gastrocnemius muscle of the test mice (*P* < 0.01), while it had no significant effect on the mRNA expression of linear *ANKRD17* (Fig. S1P). Oil red O staining demonstrated that the lipid droplet content in the liver of the LV-sh-circANKRD17 group was obviously lower than that of the control group (Fig. 6A). Compared to NC, LV-sh-circANKRD17 significantly inhibited adipocyte differentiation (*PPARγ* and *CEBPα*) (Fig. 6B, *P* < 0.01 or *P* < 0.05), fatty acid transport (*FAT1*, *FABP4*, and *FABP5*) (Fig. 6C, *P* < 0.01), fatty acid synthesis (*FASN*, *SCD*, *FADS1*), lipid oxidation (*CPT1A*) (Fig. 6D, *P* < 0.01), triglyceride synthesis (*DGAT2* and *MGAT1*) (Fig. 6E, *P* < 0.01), and lipid droplet maturation (*PLIN1*, *PLIN2*, and *PLIN3*) (Fig. 6F, *P* < 0.01). The levels of lipid metabolism-related proteins (PPARγ, CEBPα, CD36, FABP4, FABP5, FASN, LPL, CPT1A) were also significantly decreased (Fig. 6G, *P* < 0.01 o r *P* < 0.05). These results suggest that LV-sh-circANKRD17 can significantly reduce lipid metabolism in vivo.

### A novel protein encoded by circANKRD17 regulates lipid metabolism

**Fig. 7:**
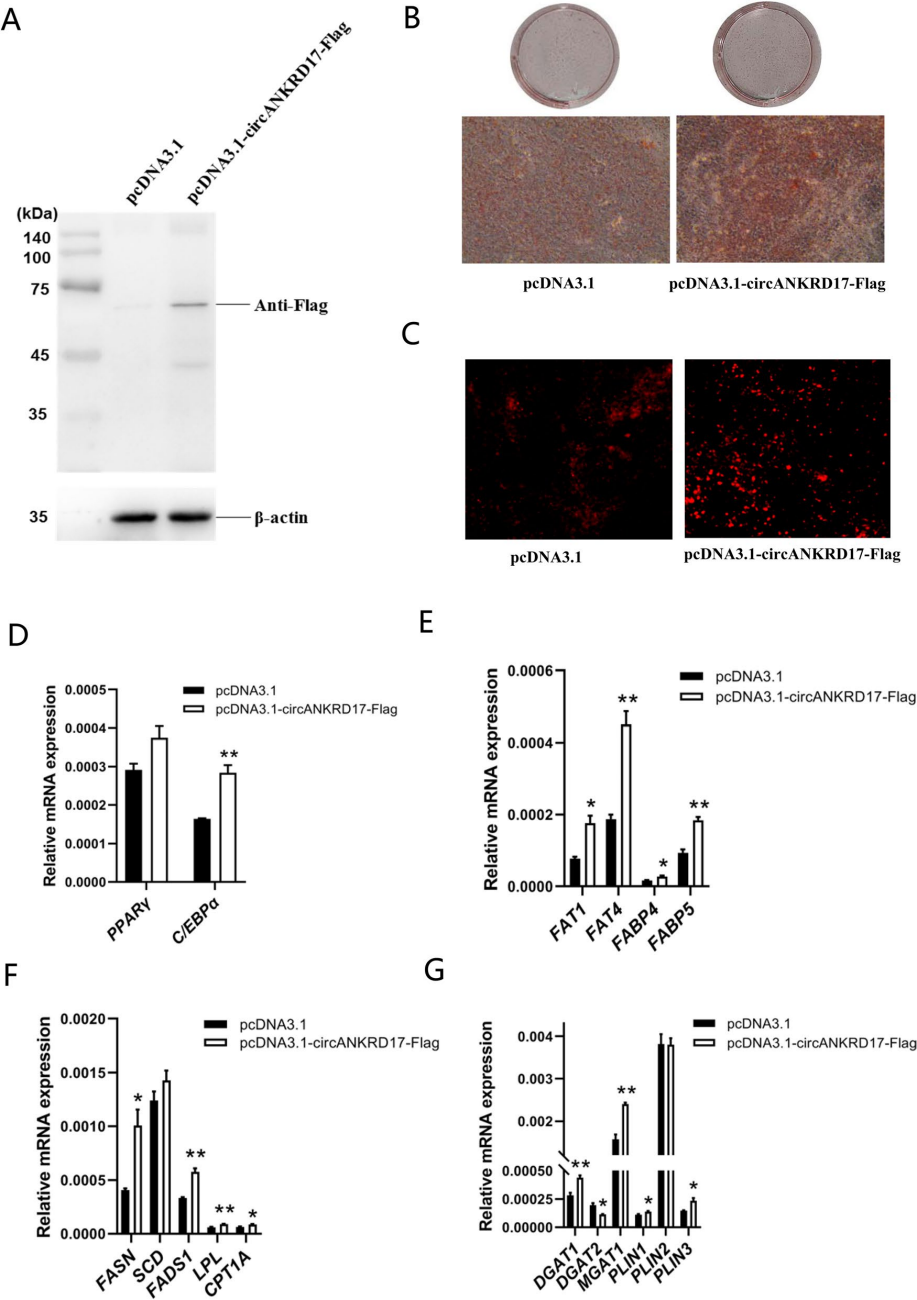
circANKRD17 encodes a novel protein to regulate lipid metabolism. **A** Levels of Flag-tagged protein in 3T3-L1 cells transfected with pCDNA3.1-circANKRD17-Flag. **B** Oil red O staining of 3T3-L1 cells transfected with either pcDNA3.1 or pCDNA3.1-circANKRD17-Flag. **C** Nile red staining of 3T3-L1 cells transfected with either pcDNA3.1 or pCDNA3.1-circANKRD17-Flag. **D** The mRNA levels of adipocyte differentiation genes. **E** The mRNA levels of fatty acid transport genes. **F** The mRNA levels of genes involved in fatty acid synthesis, hydrolysis, and oxidation. **G** The mRNA levels of triglyceride synthesis genes and lipid droplet maturation genes

We discovered that circANKRD17 contains an initiation codon and a terminator near the splice site, allowing it to encode a protein of 571 amino acids (Fig. S1C). To verify whether the predicted ORF of circANKRD17 has the ability to encode a protein, we cloned the linear sequence of the circANKRD17 ORF into the pcDNA3.1 vector, adding a 3 × Flag tag before the stop codon to construct the pCD3.1-circANKRD17-3 × Flag overexpression vector, and performed double enzyme digestion for verification (Fig. S1Q). The recombinant vector was then transfected into 3T3-L1 cells, and western blotting results showed that the protein expression of pCD3.1-circANKRD17-Flag could be detected using anti-Flag antibodies (Fig. 7A). Overexpression of pCD3.1-circANKRD17-Flag increased the number of intracellular lipid droplets, as evidenced by Oil red O staining (Fig. 7B) and Nile red staining (Fig. 7C). RT-qPCR analysis revealed that overexpression of pCD3.1-circANKRD17-Flag increased the expression of genes related to adipocyte differentiation (Fig. 7D), fatty acid transport (Fig. 7E), fatty acid synthesis and oxidation (Fig. 7F), triglyceride synthesis, and lipid droplet maturation (Fig. 7G). Western blotting was also used to verify the expression of the relevant proteins, showing that overexpression of pCD3.1-circANKRD17-Flag significantly increased the protein expression of PPARγ, CEBPα, CD36, FABP4, FABP5, FASN, LPL, and CPT1A (Fig. S1R, *P* < 0.01 or *P* < 0.05). Our results indicate that circANKRD17 can influence lipid metabolism through its encoded protein.

## Discussion

In recent years, the functions of non-coding RNAs have been extensively studied in the field of circRNA research. With continued in-depth investigation, recent studies have uncovered the coding ability of circRNAs to produce functional peptides in vivo [26]. For instance, a novel protein encoded by circ-ZNF609 was first discovered in adult myoblasts [27]. In our previous study, we demonstrated that a new protein encoded by circKANSL1L could regulate skeletal myogenesis [19]. Therefore, we combined previous circRNAome-seq data [18] and Ribo-seq data [19] to screen circRNA candidates with coding potential. We verified that circANKD17 has a cyclic structure and is differentially expressed in LT and LW breeds. Based on our single-cell sequencing data [21], we found that in IMF cells, *FABP4*, *FABP5*, *CPT1A*, and *UBC* showed significantly higher expression levels in LT piglets and were enriched in the PPAR signaling pathway. The PPAR signalling pathway plays a key role in lipid metabolism, including controlling fatty acid transport (PPARα), lipid storage (PPARγ) and glucose metabolism (PPARδ) [28]. In detail, FABP can coordinate lipid transport [24], while CPT1A can transport fatty acid from the cytosol into mitochondria [29]. In agree with our study, FABP4 was found to be highly expressed in Laiwu pigs (a local pig breed) compared to Duroc × Landrace × Yorkshire pigs [30]. Consequently, we concluded that circANKRD17 may have coding potential and could play a role in lipid metabolism through the PPAR pathway.

In comparison to lean pigs, fat-type pigs exhibit a greater capacity for adipocyte proliferation and differentiation, giving them a significant advantage in IMF deposition [31]. To further investigate the function of circANKRD17, we utilized 3T3-L1 cells as a model in vitro and C57BL/6 mice as a model in vivo, selecting a series of adipose-related genes to examine the effects on lipid metabolism through the PPAR pathway. Among these, PPARγ and CEBPα are known as key regulators of early adipogenesis [32]; *FAT1*, *FAT4*, *FABP4*, and *FABP5* are involved in fatty acid transport [33]; FASN converts carbohydrates into fatty acids for storage as triglycerides [32]; SCD is a key rate-limiting enzyme that convert saturated fatty acids to monounsaturated fatty acids [34]; and FADS1 is primarily responsible for the synthesis of polyunsaturated fatty acids [35]. Additionally, LPL serves as the rate-limiting enzyme for triglyceride degradation [36], while CPT1A facilitates the transfer of fatty acids to mitochondria for subsequent oxidative metabolic processes [25]. DGAT1 and DGAT2 are two forms of acylglycerol acyltransferases involved in triglyceride storage within adipocytes [37], with MGAT1 playing a critical role as an initiator of triglyceride synthesis and fat uptake in the body [38]. Furthermore, PLIN1, PLIN2, and PLIN3 are key genes involved in the formation of neutral lipids [39]. Our study revealed that overexpression of circANKRD17 significantly upregulated the expression of these genes and proteins, whereas interference resulted in the opposite trend. Additionally, the expression of genes related to lipolysis, such as *LPL*, also showed an upregulation trend. Generally, LPL can interact with lipoproteins, anchoring them to the vessel wall to facilitate the uptake of lipoprotein particles and the exchange of lipids between lipoproteins [40]. Typically, fatty acid synthesis, cholesterol uptake, and β-oxidation are tightly balanced [41]. CPT1A, a crucial enzyme in β-oxidation [29], may experience increased expression in response to elevated lipid metabolism demands, potentially to avoid lipid over-accumulation. However, the underlying mechanisms require further investigation. These findings demonstrate that circANKRD17 can regulate fat deposition through multiple pathways, highlighting its significant role in various aspects of lipid metabolism.

At present, the main methods for verifying the coding ability of circRNAs include adding tagged proteins to the ORF coding region of circRNAs to detect the expression of fusion proteins, or inserting the ORF coding region of circRNAs into a dual-luciferase coding sequence to determine whether the ORF coding region is readable [42]. In this study, to verify the coding ability of circANKRD17, we constructed the vector pcDNA3.1-circANKRD17-Flag, which allowed for the fusion expression of the ORF of circANKRD17 with a Flag-tagged protein. We performed western blot analysis using a Flag-tagged antibody and detected a protein of the expected size for the circANKRD17-Flag fusion, thereby confirming the coding ability of circANKRD17. Cap-independent translation of circRNAs driven by internal ribosome entry site (IRES) or N^6^-methyladenosine (m^6^A)-containing short sequence [17]. However, the translation mechanism of circANKRD17 remains unclear, and it constitutes a key research objective for our subsequent experiments. One might argue that the protein encoded by circANKRD17 specifically interacts with the PPAR pathway to influence intramuscular fat metabolism and that the proposed molecular mechanisms underlying this interaction are based on the findings of this investigation.

## Conclusion

In summary, our study identifies circANKRD17 as a key regulator of lipid metabolism, with its encoded protein significantly contributing to adipogenesis and lipid metabolism. These findings provide new insights into the metabolic characteristics of pig breeds with higher intramuscular fat content.

## Supplementary Information

Additional file 1: Table S1A. The primer sequences used in our study. Table S1B. The protein-coding circRNA candidates identified with the ribosome profiling sequencing. Table S1C. Lipid metabolites identified in porcine longissimus dorsi muscles. Table S1D. Differential lipid metabolites in porcine longissimus dorsi muscles between LT and LW piglets.

Additional file 2: Fig. S1A. RT-PCR and Sanger sequencing to validate the junction sites of candidate circRNAs. Fig. S1B. Relative expression levels of candidate circRNAs between LT and LW. Fig. S1C. Schematic diagram illustrating the translation potential of circANKRD17. Fig. S1D. Schematic representation of the open reading frame of human circANKRD17. Fig. S1E. RT-PCR amplification of the full-length sequence of murine-derived circANKRD17. Fig. S1F. Diagram of the pCD2.1-ciR overexpression vector. Fig. S1G. Identification of the OE-circANKRD17 overexpression plasmid by double digestion. Fig. S1H. Expression levels of circANKRD17 and ANKRD17 mRNA in 3T3-L1 cells transfected with circANKRD17 overexpression vectors. Fig. S1I. Knockdown efficiency of two siRNA sequences targeting circANKRD17 in 3T3-L1 cells. Different lowercase letters indicate *P *< 0.05. Fig. S1J. Nile red staining fluorescence and triglyceride content assay of 3T3-L1 cells transfected with circANKRD17 overexpression vectors. Fig. S1K. Nile red fluorescence and triglyceride content assay of 3T3-L1 cells transfected with circANKRD17 knockdown vectors. Fig. S1L. Diagram of the pshRNA-copGFP Lentivector. Fig. S1M. Colony PCR and Sanger sequencing results of the recombinant plasmid sh-circANKRD17. The horizontal segment represents the inserted interfering sequence. Fig. S1N. Diluted lentivirus supernatant was transfected into 293T cells, and the titer of the lentivirus was determined by observing green fluorescent protein under a fluorescence microscope. Fig. S1O. Oil red O staining of mouse livers infected with either LV-sh-circANKRD17 lentivirus or control in the standard diet groups. Fig. S1P. Expression of circANKRD17 and mANKRD17 mRNA in LV-sh-circANKRD17-transfected 293T cells. Fig. S1Q. Diagram of the pCDNA3.1 vector and identification of the pCDNA3.1-circANKRD17-3×Flag overexpression plasmid by double digestion. Fig. S1R. Protein levels related to lipid metabolism after treatment with either the negative control or pCDNA3.1-circANKRD17-Flag.

## Data Availability

Not applicable.

## Abbreviations

ACar: Acylcarnitines
circRNA: Circular RNA
FISH: Fluorescence in situ hybridization
gDNA: Genomic DNA
IMF: Intramuscular fat
IRES: Internal ribosome entry site
lncRNA: Long non-coding RNA
miRNA: MicroRNA
ncRNA: Non-coding RNA
ORF: Open reading frame
PC: Phosphatidylcholine
PE: Phosphatidylethanolamine
TAG: Triacylglycerol
