# Supplementary material for: A novel protein encoded by porcine circANKRD17 activates the PPAR pathway to regulate intramuscular fat metabolism
Source: J Anim Sci Biotechnol. 2025 Feb 5;16:19. doi: 10.1186/s40104-025-01153-5 (PMC11796117; doi:10.1186/s40104-025-01153-5)
Supplement: Supplementary file 2 — Additional file 2: Fig. S1A. RT-PCR and Sanger sequencing to validate the junction sites of candidate circRNAs. Fig. S1B. Relative expression levels of candidate circRNAs between LT and LW. Fig. S1C. Schematic diagram illustrating the translation potential of circANKRD17. Fig. S1D. Schematic representation of the open reading frame of human circANKRD17. Fig. S1E. RT-PCR amplification of the full-length sequence of murine-derived circANKRD17. Fig. S1F. Diagram of the pCD2.1-ciR overexpression vector. Fig. S1G. Identification of the OE-circANKRD17 overexpression plasmid by double digestion. Fig. S1H. Expression levels of circANKRD17 and ANKRD17 mRNA in 3T3-L1 cells transfected with circANKRD17 overexpression vectors. Fig. S1I. Knockdown efficiency of two siRNA sequences targeting circANKRD17 in 3T3-L1 cells. Different lowercase letters indicate P < 0.05. Fig. S1J. Nile red staining fluorescence and triglyceride content assay of 3T3-L1 cells transfected with circANKRD17 overexpression vectors. Fig. S1K. Nile red fluorescence and triglyceride content assay of 3T3-L1 cells transfected with circANKRD17 knockdown vectors. Fig. S1L. Diagram of the pshRNA-copGFP Lentivector. Fig. S1M. Colony PCR and Sanger sequencing results of the recombinant plasmid sh-circANKRD17. The horizontal segment represents the inserted interfering sequence. Fig. S1N. Diluted lentivirus supernatant was transfected into 293T cells, and the titer of the lentivirus was determined by observing green fluorescent protein under a fluorescence microscope. Fig. S1O. Oil red O staining of mouse livers infected with either LV-sh-circANKRD17 lentivirus or control in the standard diet groups. Fig. S1P. Expression of circANKRD17 and mANKRD17 mRNA in LV-sh-circANKRD17-transfected 293T cells. Fig. S1Q. Diagram of the pCDNA3.1 vector and identification of the pCDNA3.1-circANKRD17-3×Flag overexpression plasmid by double digestion. Fig. S1R. Protein levels related to lipid metabolism after treatment with either the n [file 40104_2025_1153_MOESM2_ESM.pdf]

A

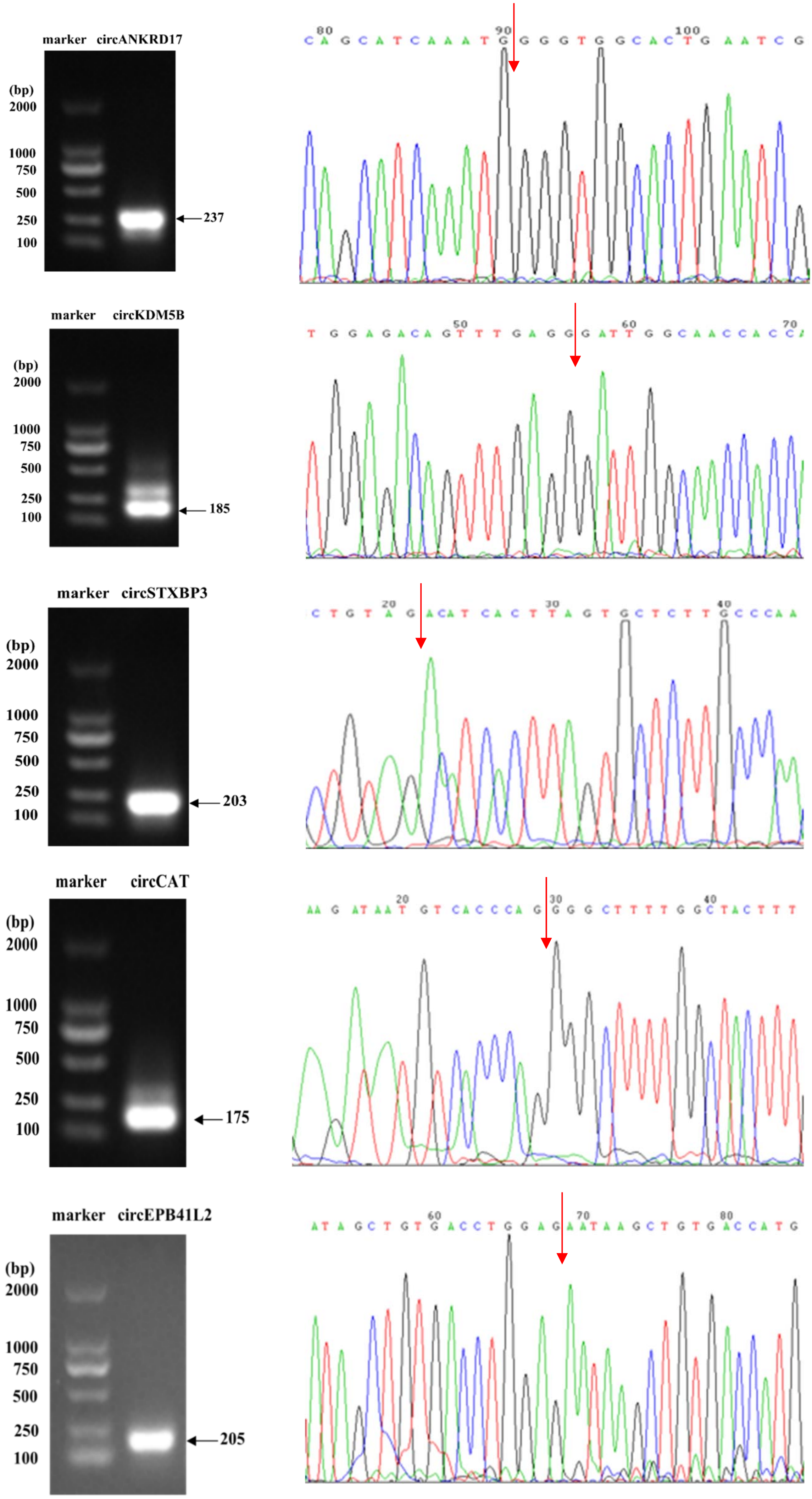

marker
circANKRD17
(bp)
2000
1000
750
500
250
100
237
marker
circKDM5B
(bp)
2000
1000
750
500
250
100
185
marker
circSTXBP3
(bp)
2000
1000
750
500
250
100
203
marker
circCAT
(bp)
2000
1000
750
500
250
100
175
marker
circEPB41L2
(bp)
2000
1000
750
500
250
100
205

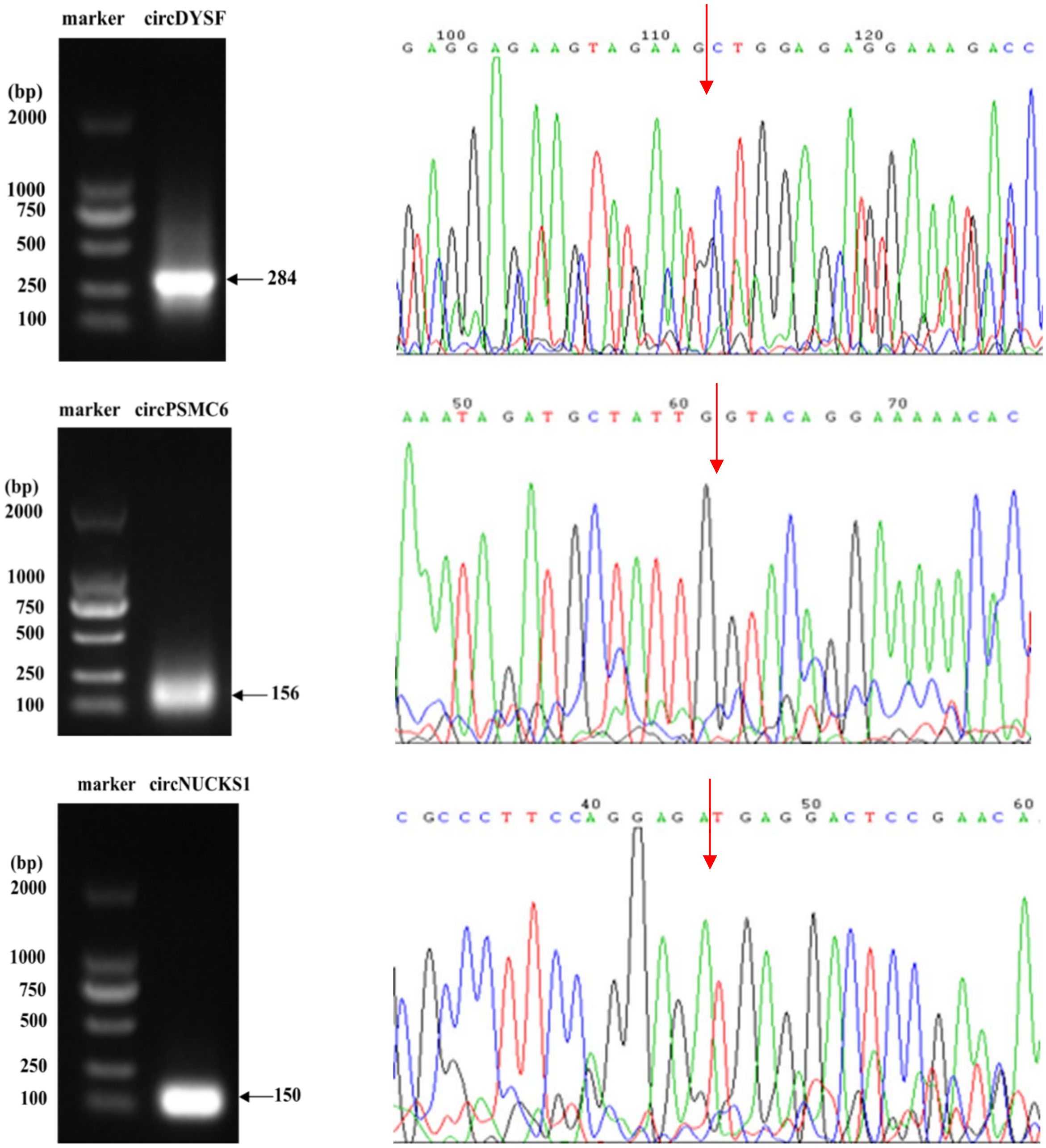
marker
circDYSF
(bp)
2000
1000
750
500
250
100
284
marker
circPSMC6
(bp)
2000
1000
750
500
250
100
156
marker
circNUCKS1
(bp)
2000
1000
750
500
250
100
150

B

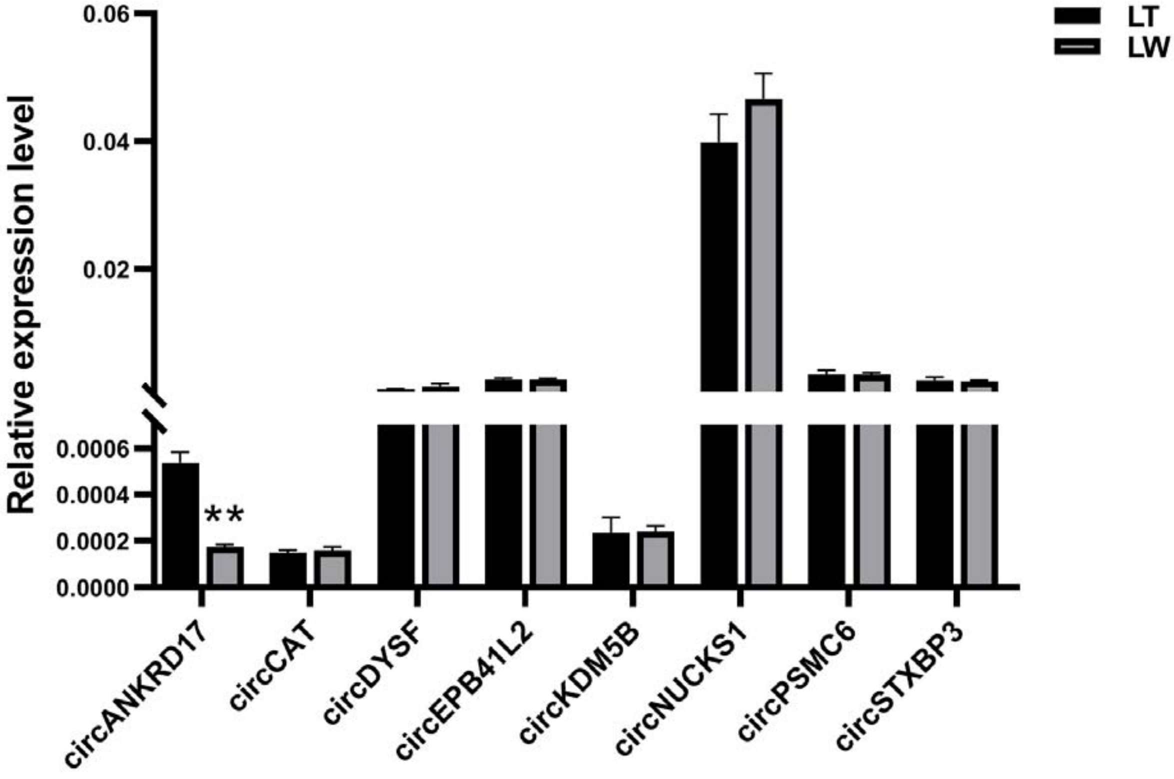
Relative expression level
0.06
0.04
0.02
0.0006
0.0004
0.0002
0.0000
LT
LW
**
circANKRD17
circCAT
circDYSF
circEPB41L2
circKDM5B
circNUCKS1
circPSMC6
circSTXBP3

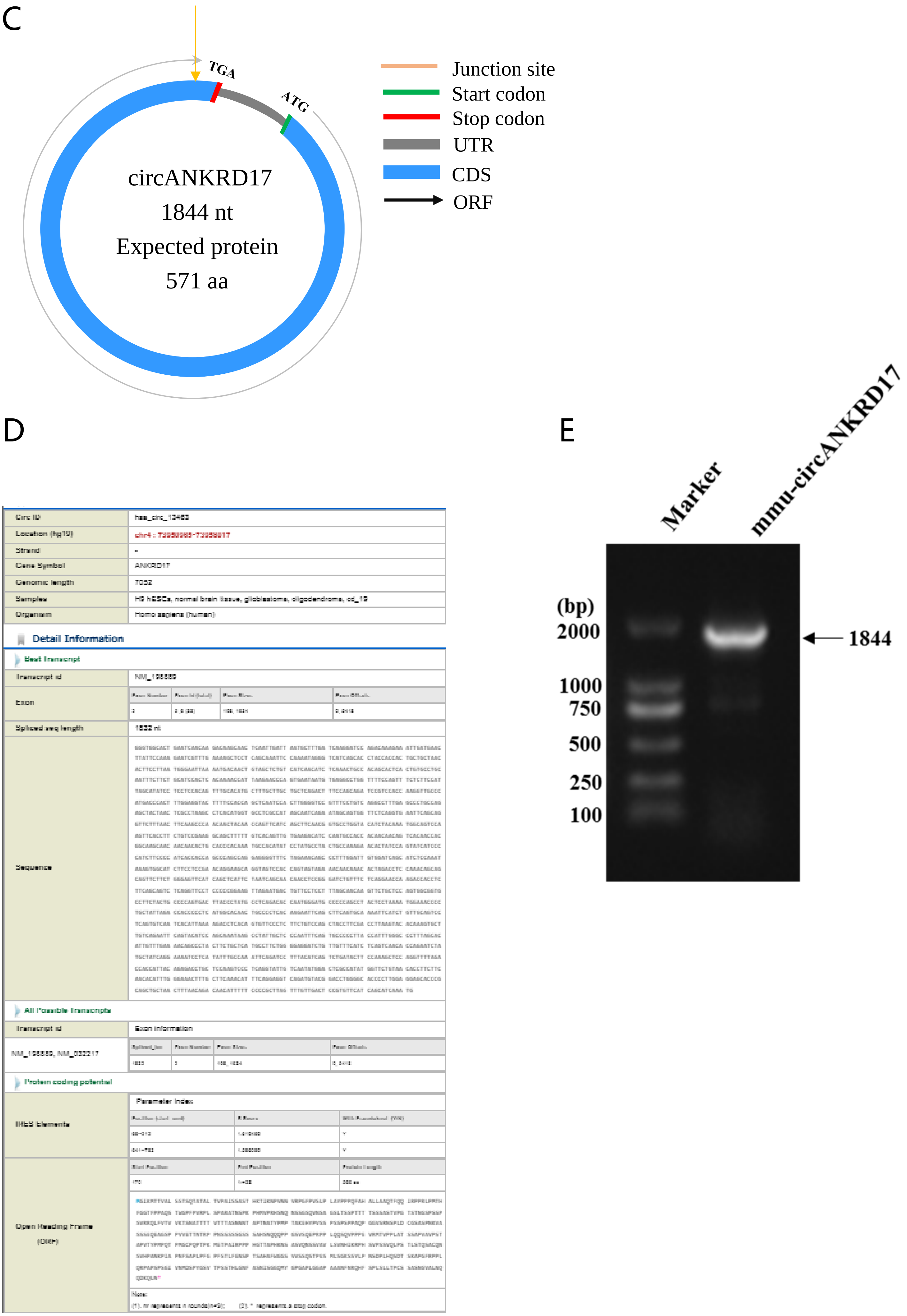
C
TGA
ATG
circANKRD17
1844 nt
Expected protein
571 aa
Junction site
Start codon
Stop codon
UTR
CDS
ORF
D
Circ ID
Location (hg19)
Strand
Gene Symbol
ANKRD17
Genomic length
Samples
Organism
Homo sapiens (human)
Detail Information
Best Transcript
Transcript id
Exon
Spliced seq length
Sequence
All Possible Transcripts
Transcript id
Exon information
Protein coding potential
Parameter Index
IRES Elements
Open Reading Frame
(ORF)
Note:
E
Marker
mmu-circANKRD17
(bp)
2000
1000
750
500
250
100
1844

F

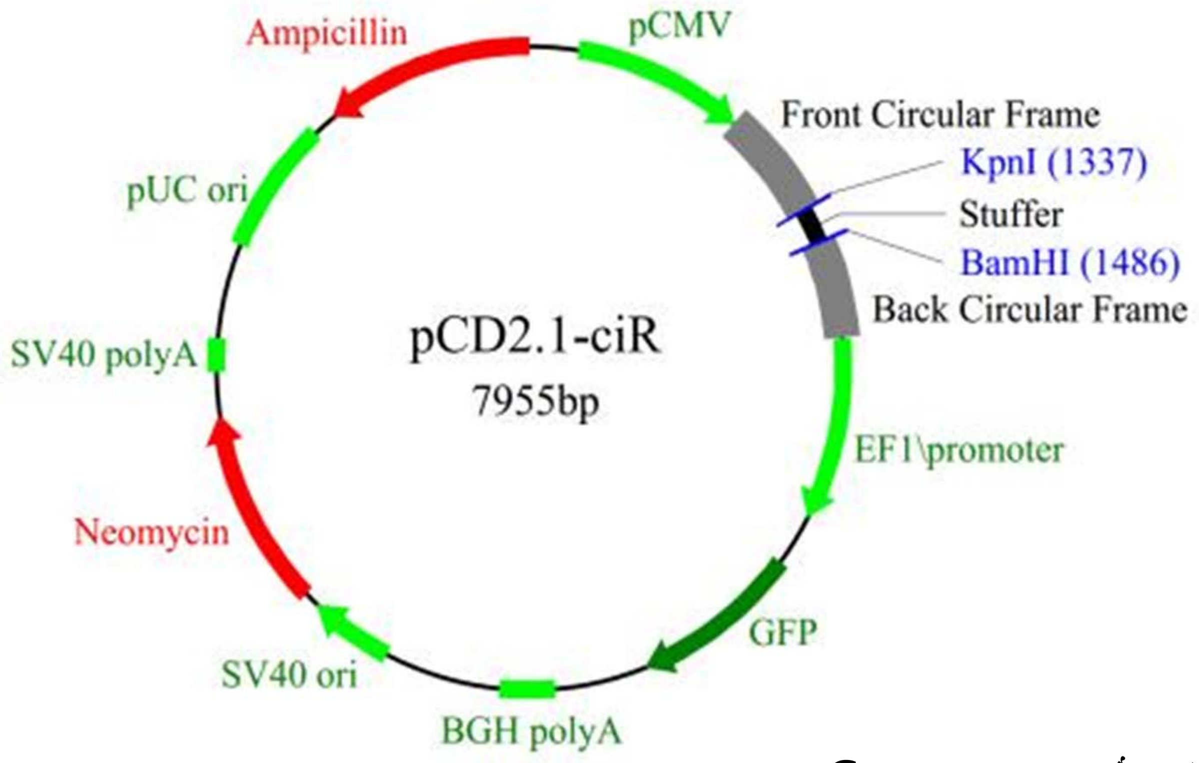
Ampicillin
pCMV
Front Circular Frame
KpnI (1337)
Stuffer
BamHI (1486)
Back Circular Frame
pUC ori
SV40 polyA
pCD2.1-ciR
7955bp
EF1\promoter
Neomycin
GFP
SV40 ori
BGH polyA

G

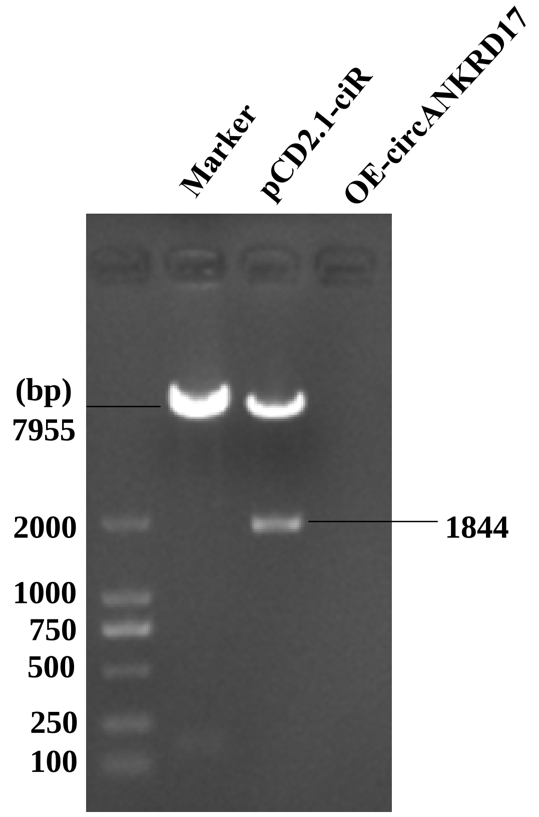
Marker
pCD2.1-ciR
OE-circANKRD17
(bp)
7955
2000
1000
750
500
250
100
1844

H

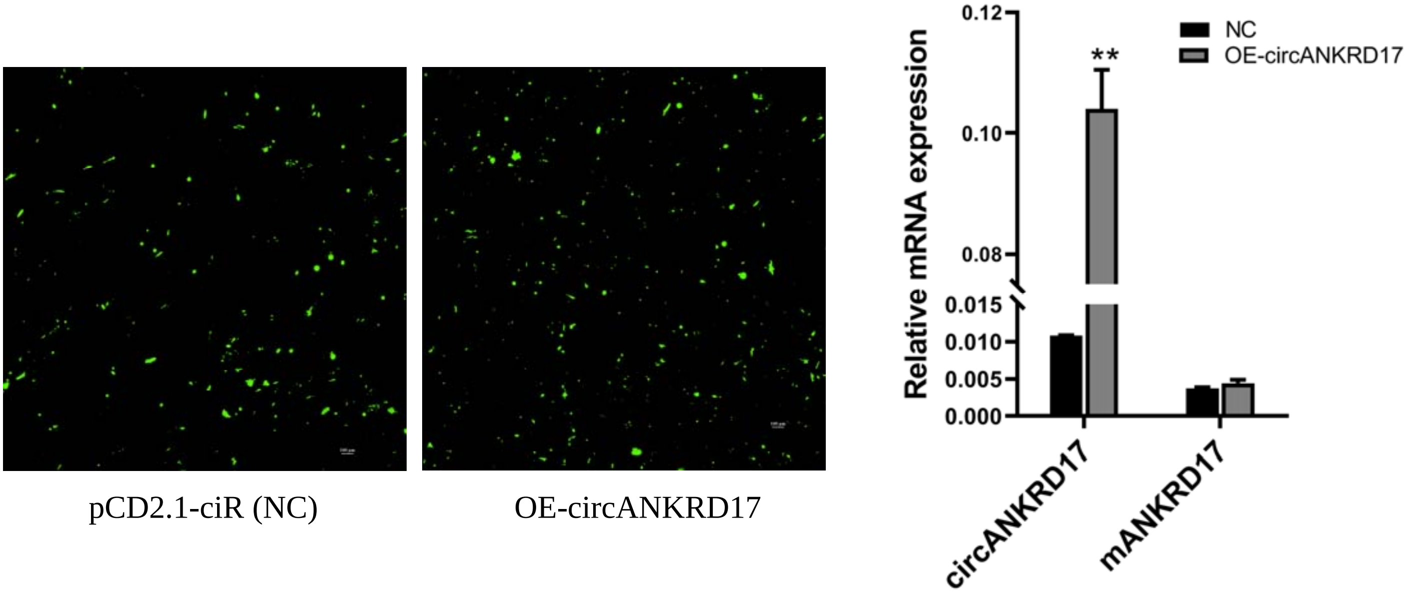
pCD2.1-ciR (NC)
OE-circANKRD17
Relative mRNA expression
0.12
0.10
0.08
0.015
0.010
0.005
0.000
**
NC
OE-circANKRD17
circANKRD17
mANKRD17

I

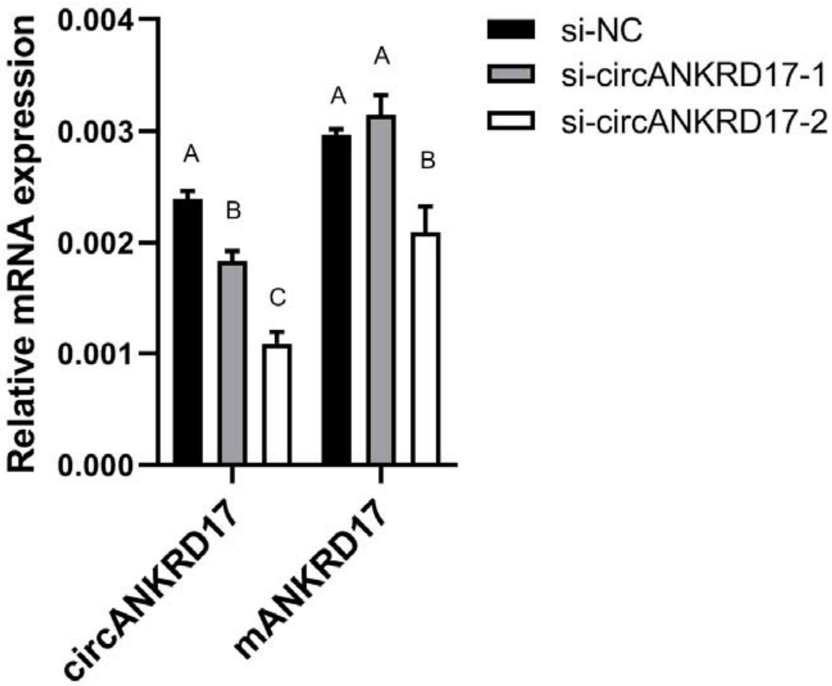

Relative mRNA expression
0.004
0.003
0.002
0.001
0.000
si-NC
si-circANKRD17-1
si-circANKRD17-2
A
B
C
A
A
B
circANKRD17
mANKRD17

J

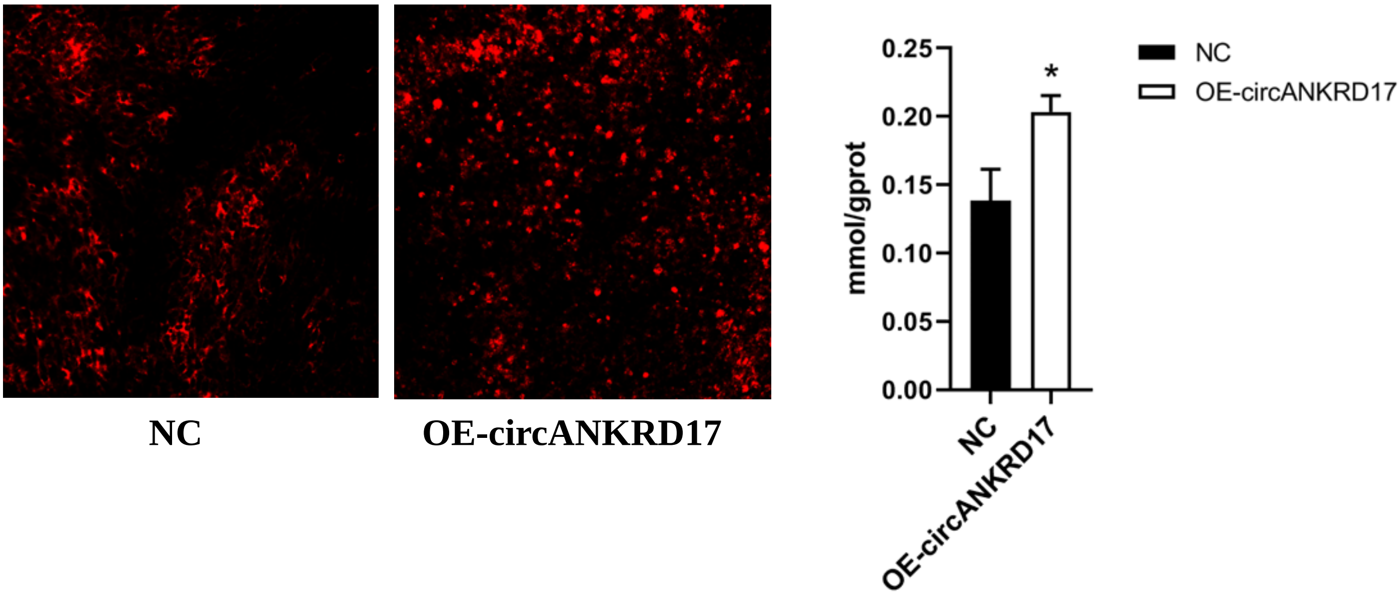

NC
OE-circANKRD17
mmol/gprot
0.25
0.20
0.15
0.10
0.05
0.00
*
NC
OE-circANKRD17

K

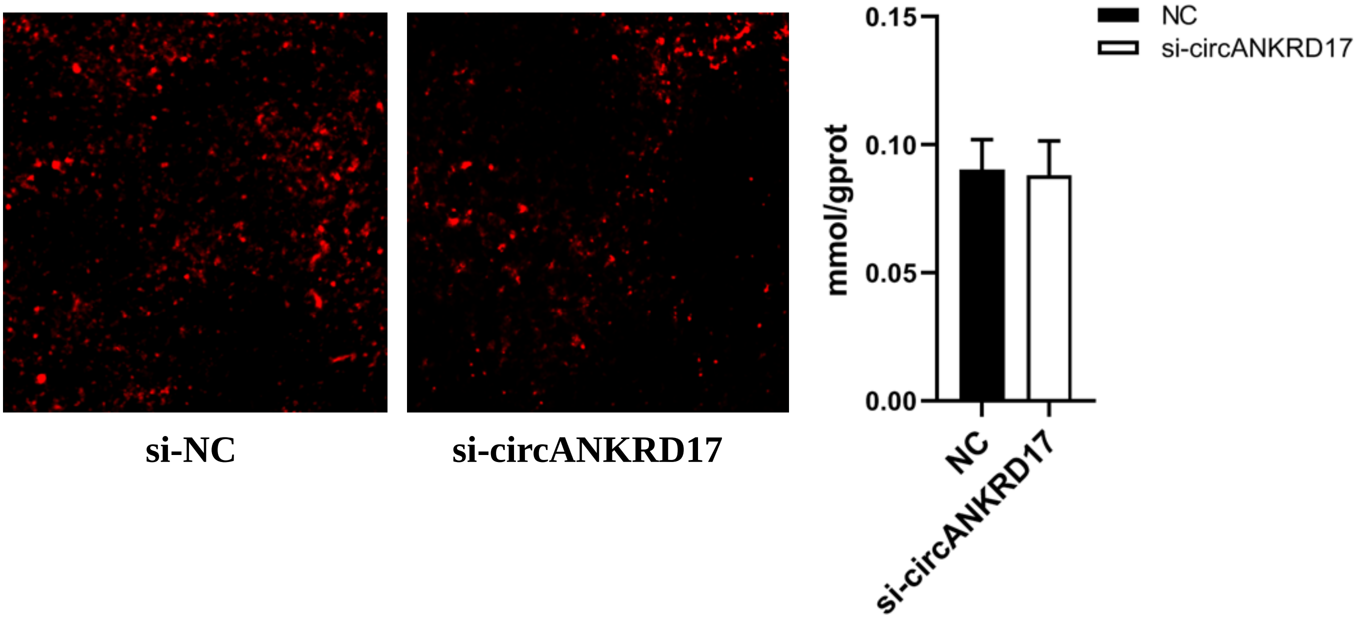

si-NC
si-circANKRD17
mmol/gprot
0.15
0.10
0.05
0.00
NC
si-circANKRD17

L

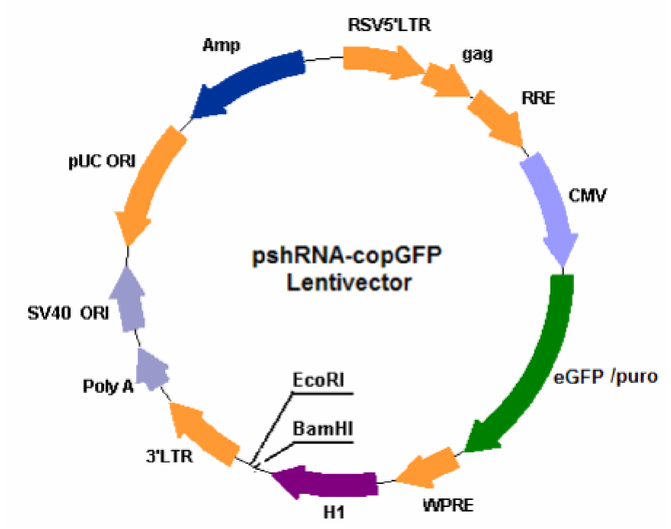
pshRNA-copGFP
Lentivector
RSV5'LTR
gag
RRE
CMV
eGFP /puro
WPRE
H1
BamHI
EcoRI
3'LTR
Poly A
SV40 ORI
pUC ORI
Amp

M

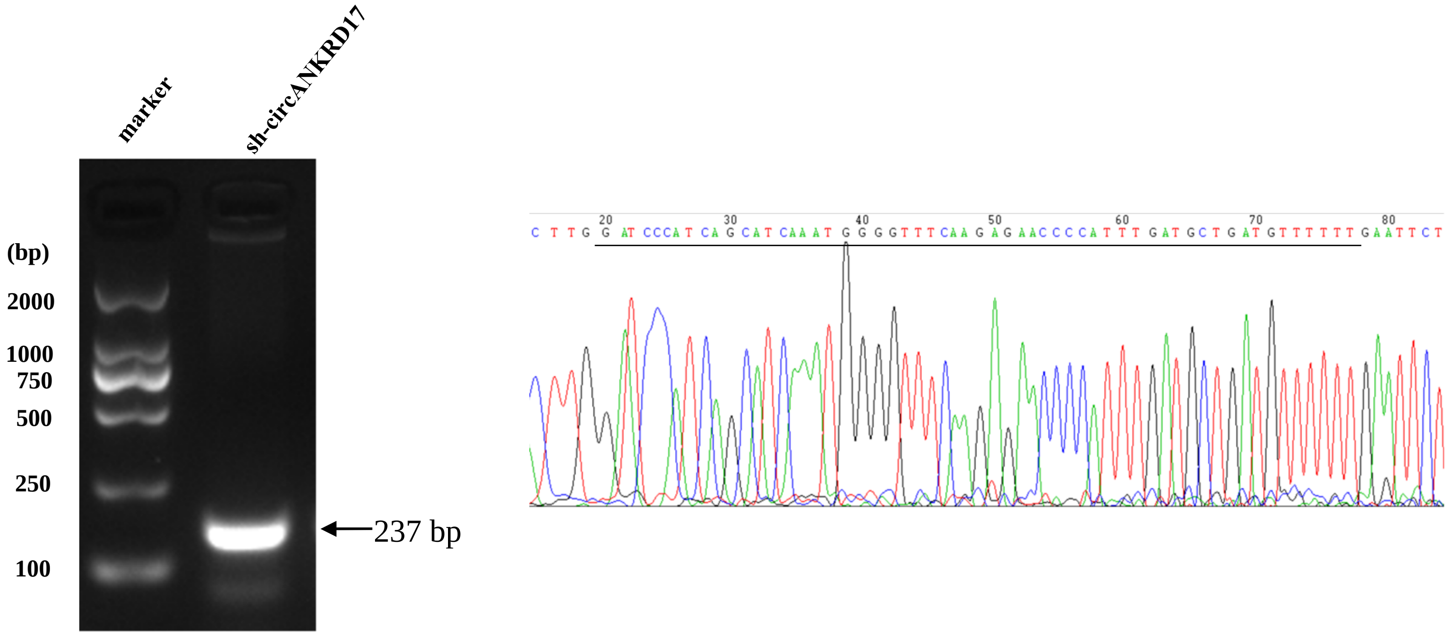
marker
sh-circANKRD17
(bp)
2000
1000
750
500
250
100
237 bp
20 30 40 50 60 70 80
C T T G G AT CCCAT CA G CAT CAAAT G G G GTTTCAA G AG AACCCCATTT GAT GCT G AT GTTTTTT GAATTCT

N

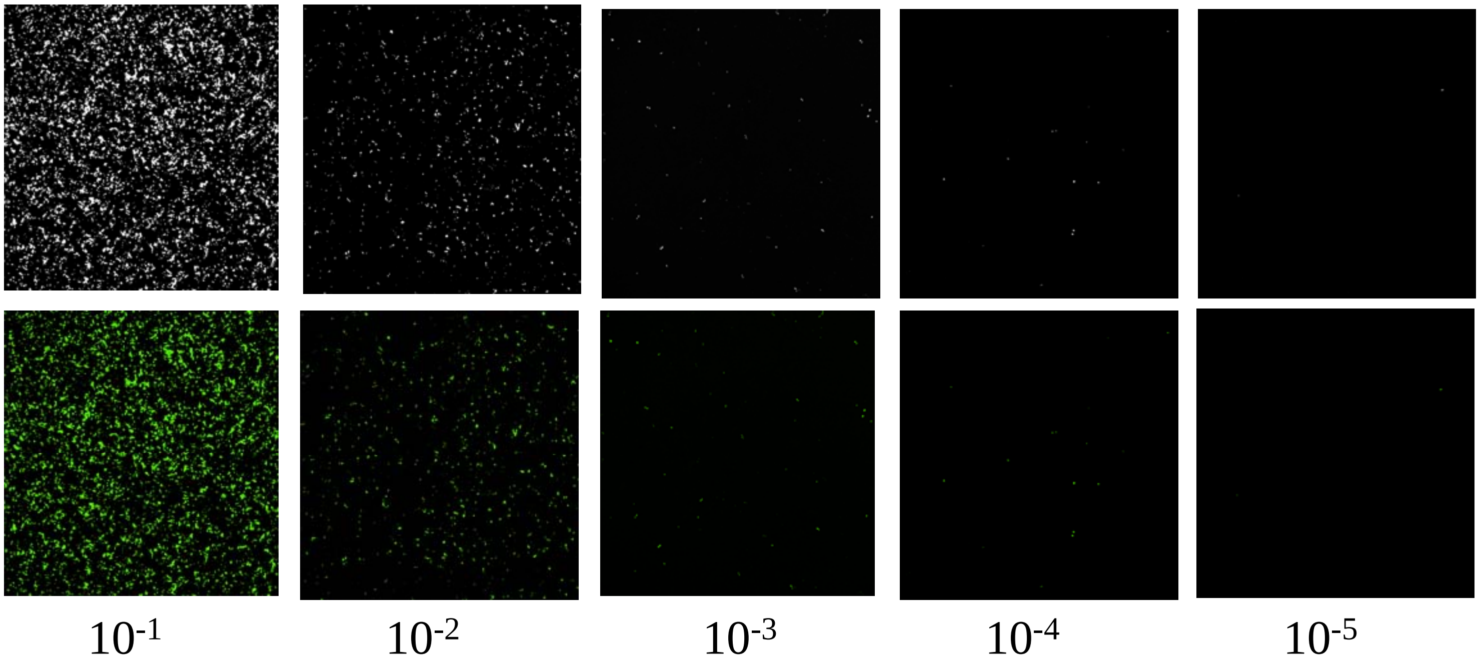
10^{-1}
10^{-2}
10^{-3}
10^{-4}
10^{-5}

O

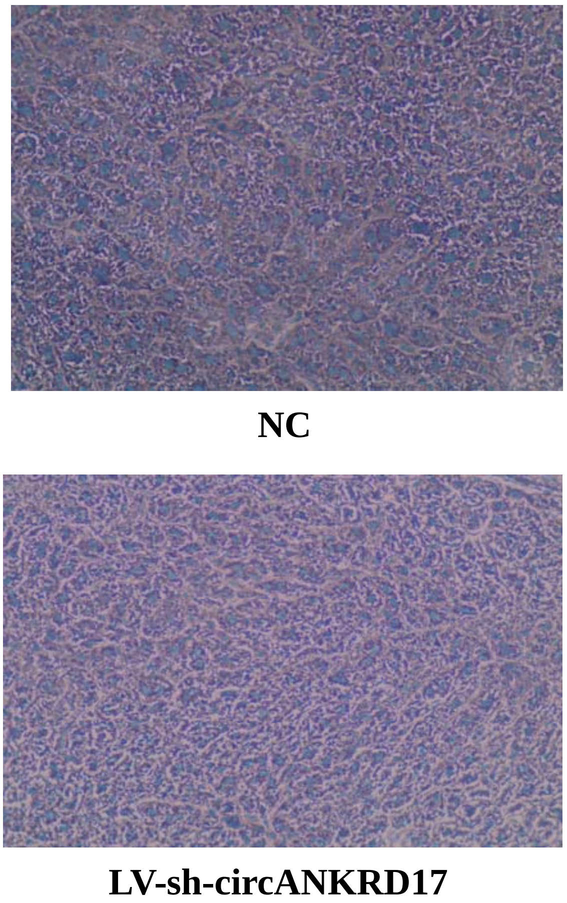
NC
LV-sh-circANKRD17

P

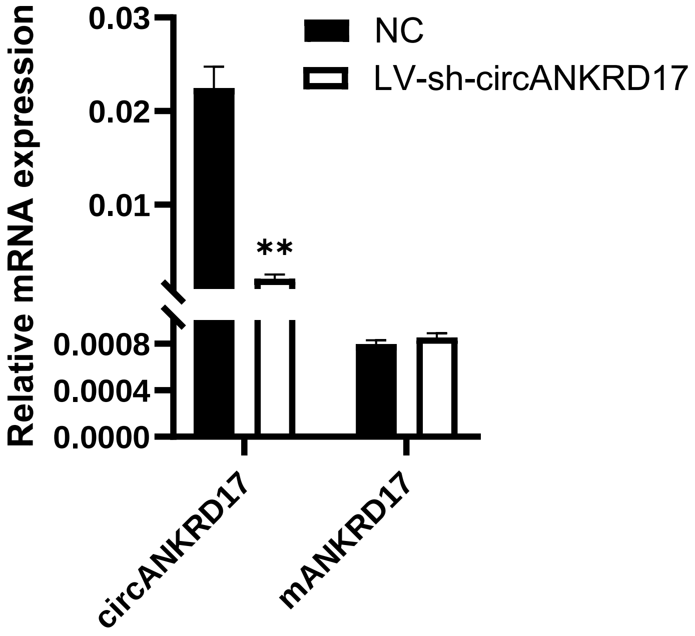
Relative mRNA expression
0.03
0.02
0.01
0.0008
0.0004
0.0000
NC
LV-sh-circANKRD17
**
circANKRD17
mANKRD17

Q

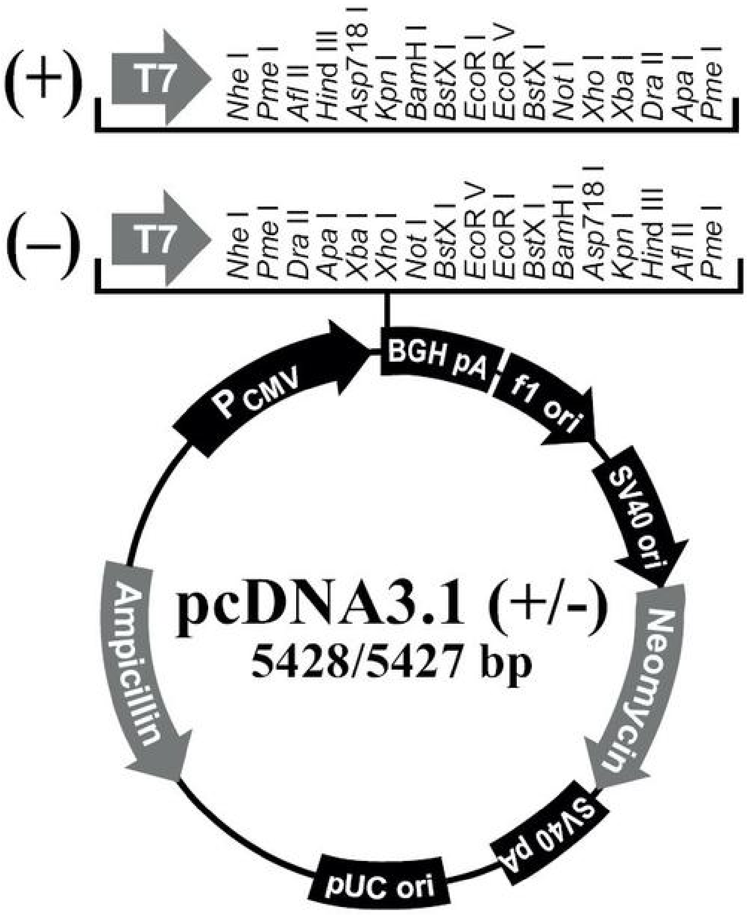
(+)
T7
Nhe I
Pme I
Afl II
Hind III
Asp718 I
Kpn I
BamH I
BstX I
EcoR I
EcoR V
BstX I
Not I
Xho I
Xba I
Dra II
Apa I
Pme I
(–)
T7
Nhe I
Pme I
Dra II
Apa I
Xba I
Xho I
Not I
BstX I
EcoR V
EcoR I
BstX I
BamH I
Asp718 I
Kpn I
Hind III
Afl II
Pme I
P CMV
BGH pA
f1 ori
SV40 ori
Neomycin
SV40 pA
pUC ori
Ampicillin
pcDNA3.1 (+/-)
5428/5427 bp

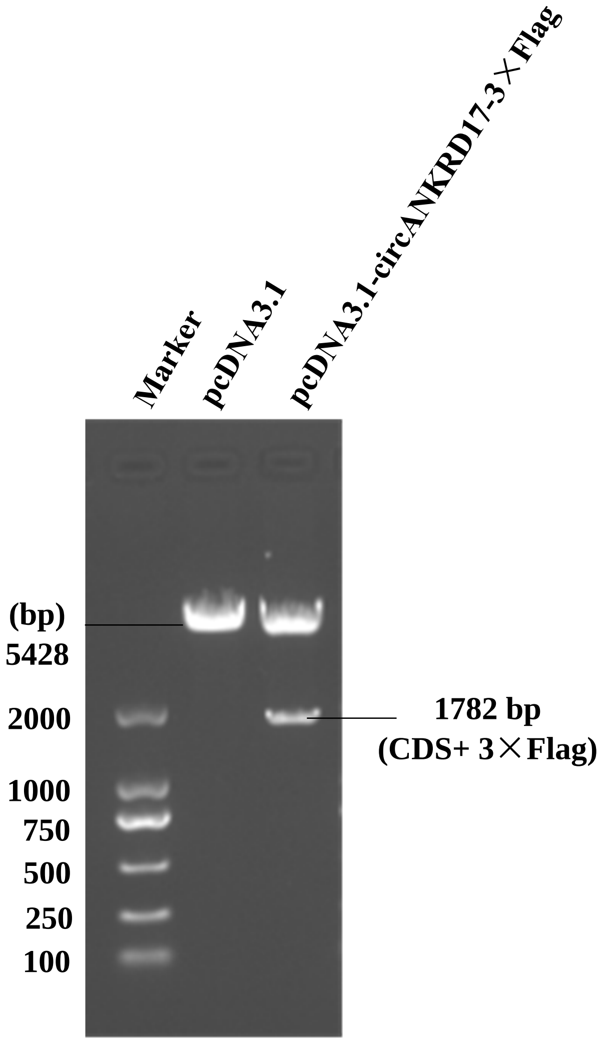
Marker
pcDNA3.1
pcDNA3.1-circANKRD17-3×Flag
(bp)
5428
2000
1000
750
500
250
100
1782 bp
(CDS+ 3×Flag)

R

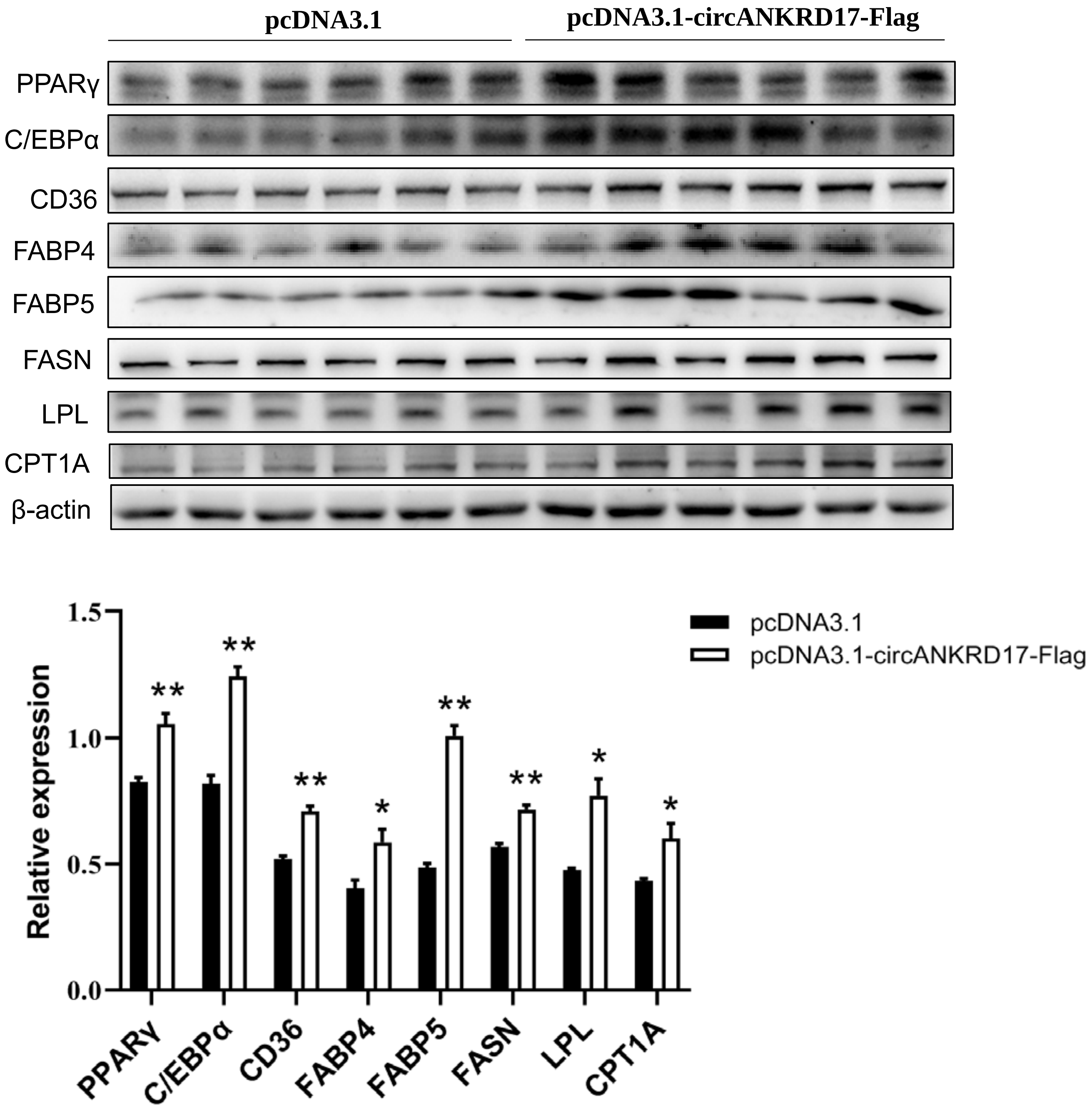
pcDNA3.1
pcDNA3.1-circANKRD17-Flag
PPARγ
C/EBPα
CD36
FABP4
FABP5
FASN
LPL
CPT1A
β-actin
Relative expression
1.5
1.0
0.5
0.0
**
**
**
*
**
**
*
*
PPARγ
C/EBPα
CD36
FABP4
FABP5
FASN
LPL
CPT1A
pcDNA3.1
pcDNA3.1-circANKRD17-Flag
